# Supplementary material for: Genetically Raised Circulating Bilirubin Levels and Risk of Ten Cancers: A Mendelian Randomization Study
Source: Cells. 2021 Feb 15;10(2):394. doi: 10.3390/cells10020394 (PMC7918902; doi:10.3390/cells10020394)
Supplement: Supplementary file 1 [file cells-10-00394-s001.pdf]

Genetically raised circulating bilirubin levels and risk of ten cancers: a Mendelian randomization study

Nazlisadat Seyed Khoei, Robert Carreras-Torres et al.

Tables S1 Summary statistics for genetic association of total bilirubin levels and risk of ten cancers

Tables S2 Results of sensitivity Mendelian randomization methods for total bilirubin levels and risk of ten cancers

Figures S1 Scatter plots depicting the genetic associations between bilirubin levels and risk of pancreatic cancer and renal cell carcinoma, overall and sex subgroups

Figures S2 Scatter plots depicting the genetic associations between bilirubin levels and lung cancer risk, overall, smoking subgroups, and histological subtypes

Figures S3 Scatter plots depicting the genetic associations between bilirubin levels and risk of ovarian, endometrial, and breast cancers, overall and subtypes

Figures S4 Scatter plots depicting the genetic association between bilirubin levels and prostate cancer risk.

Figures S5 Scatter plots depicting the genetic associations between bilirubin levels and risk of Hodgkin's lymphoma, melanoma, and neuroblastoma.

Funding Sources and Acknowledgements

Table S1 | Summary statistics for genetic association of total bilirubin levels and risk of ten cancers

					Total bilirubin			Pancreatic cancer overall		Pancreatic cancer in men		Pancreatic cancer in women		Renal cell cancer overall		Renal cell cancer in men		Renal cell cancer in women		Lung cancer overall		Lung cancer overall in ever smokers		Lung cancer overall in never smokers	
SNPs	Chromosome	Base pair position	Effect allele	Reference allele	Beta	SE	Explained variance (%)	Beta	SE	Beta	SE	Beta	SE	Beta	SE	Beta	SE	Beta	SE	Beta	SE	Beta	SE	Beta	SE
rs2375279	1	25541931	C	T	0.022	0.004	1.29E-04	-0.031	0.033	0.008	0.045	-0.078	0.049	0.006	0.025	-0.026	0.047	0.062	0.060	-0.007	0.017	-0.001	0.021	0.028	0.051
rs17513135	1	40035686	C	T	0.018	0.003	1.17E-04	-0.031	0.028	-0.049	0.038	-0.007	0.042	0.012	0.021	0.065	0.040	-0.025	0.051	0.006	0.014	-0.002	0.017	0.073	0.041
rs6682423	1	63171063	T	C	0.021	0.003	1.98E-04	-0.027	0.025	-0.034	0.034	-0.019	0.037	-0.010	0.019	0.009	0.037	0.041	0.046	-0.012	0.012	-0.011	0.016	-0.019	0.038
rs1762486	1	107627697	A	G	0.020	0.003	1.78E-04	-0.044	0.026	-0.048	0.035	-0.043	0.038	0.000	0.020	-0.017	0.038	0.037	0.048	-0.001	0.013	-0.006	0.016	-0.024	0.038
rs61812598	1	154420087	A	G	0.016	0.003	1.24E-04	-0.010	0.024	0.012	0.033	-0.034	0.036	-0.014	0.019	-0.042	0.036	-0.042	0.045	0.011	0.012	0.016	0.015	-0.024	0.036
rs857725	1	158607935	T	G	0.033	0.003	4.33E-04	-0.037	0.027	-0.040	0.036	-0.037	0.040	-0.017	0.020	-0.046	0.038	-0.009	0.049	-0.026	0.013	-0.008	0.016	-0.086	0.040
rs788644	1	202256962	G	T	0.018	0.003	1.56E-04	0.030	0.024	-0.004	0.032	0.070	0.036	-0.024	0.018	0.014	0.034	-0.009	0.044	-0.006	0.012	0.008	0.015	-0.015	0.035
rs1874121	1	220969049	T	C	0.023	0.003	2.47E-04	0.034	0.026	-0.002	0.035	0.076	0.038	0.000	0.020	-0.062	0.038	0.020	0.048	-0.002	0.013	-0.002	0.017	-0.017	0.039
rs556107	1	234853059	T	C	0.021	0.003	2.28E-04	-0.038	0.024	-0.043	0.032	-0.032	0.036	0.005	0.018	-0.013	0.036	0.017	0.044	0.007	0.012	-0.003	0.015	0.036	0.035
rs13030095	2	26026598	A	G	0.015	0.003	9.79E-05	-0.016	0.026	0.012	0.036	-0.047	0.039	-0.004	0.020	-0.028	0.039	-0.012	0.049	0.014	0.013	-0.007	0.017	0.041	0.039
rs2053799	2	32883197	G	A	0.015	0.003	1.03E-04	0.021	0.024	0.013	0.033	0.025	0.036	0.012	0.019	-0.018	0.036	0.085	0.044	0.012	0.012	0.027	0.015	-0.056	0.036
rs4671605	2	64887382	T	C	0.021	0.003	1.65E-04	0.034	0.027	-0.005	0.037	0.077	0.040	0.040	0.021	0.044	0.039	0.035	0.050	0.018	0.013	0.018	0.017	-0.026	0.039
rs6734238	2	113841030	A	G	0.023	0.003	2.46E-04	-0.002	0.024	0.037	0.033	-0.046	0.036	-0.022	0.019	0.005	0.036	-0.006	0.045	-0.008	0.012	-0.016	0.015	0.045	0.036
rs1047891	2	211540507	A	C	0.023	0.003	2.36E-04	-0.013	0.026	-0.020	0.036	-0.006	0.040	0.009	0.021	-0.029	0.040	0.051	0.050	-0.001	0.013	0.002	0.016	-0.002	0.038
rs6731997	2	232560411	G	A	0.017	0.003	9.89E-05	0.055	0.029	0.046	0.039	0.069	0.043	-0.029	0.022	0.014	0.041	0.006	0.051	-0.012	0.014	-0.030	0.018	0.063	0.043
rs4973588	2	233834975	A	G	0.035	0.004	2.97E-04	0.012	0.033	0.034	0.044	-0.014	0.049	-0.017	0.025	-0.023	0.048	-0.057	0.060	0.012	0.016	-0.002	0.020	0.097	0.047
**rs6431625**	**2**	**234637912**	**C**	**T**	**0.598**	**0.002**	**0.169**	**0.014**	**0.024**	**0.036**	**0.033**	**-0.013**	**0.036**	**0.005**	**0.019**	**0.003**	**0.037**	**0.059**	**0.046**	**-0.012**	**0.012**	**-0.028**	**0.015**	**-0.004**	**0.036**
rs10929023	2	235169902	G	A	0.023	0.003	2.14E-04	-0.001	0.026	0.020	0.036	-0.029	0.039	0.040	0.020	-0.044	0.039	0.034	0.050	0.007	0.013	0.009	0.017	-0.021	0.039
rs2267846	3	48556339	A	G	0.017	0.003	1.08E-04	0.059	0.027	0.083	0.036	0.029	0.040	0.014	0.020	0.057	0.038	-0.023	0.049	0.018	0.013	0.020	0.017	-0.013	0.041
rs9826148	3	114464858	C	T	0.026	0.004	1.28E-04	-0.066	0.037	-0.023	0.049	-0.118	0.055	0.033	0.028	0.007	0.052	0.042	0.066	-0.001	0.019	-0.023	0.023	0.053	0.054
rs6779903	3	135720851	T	G	0.019	0.003	1.44E-04	-0.016	0.027	-0.027	0.036	-0.003	0.040	-0.025	0.020	-0.029	0.039	0.032	0.048	0.024	0.013	0.012	0.017	0.076	0.039
rs1052618	3	136574501	A	G	0.028	0.003	3.45E-04	-0.021	0.026	0.008	0.035	-0.053	0.038	-0.025	0.020	-0.064	0.038	0.039	0.046	0.011	0.013	0.011	0.016	0.043	0.038
rs1482852	3	156798294	A	G	0.017	0.003	1.32E-04	0.035	0.024	0.012	0.033	0.068	0.036	-0.004	0.019	0.006	0.035	-0.021	0.043	-0.002	0.012	0.013	0.015	-0.055	0.036
rs61791066	3	170713380	C	T	0.031	0.004	2.06E-04	0.011	0.034	0.033	0.047	-0.012	0.051	0.017	0.026	-0.083	0.051	0.077	0.063	-0.025	0.017	-0.025	0.021	-0.088	0.052
rs11917973	3	195838613	T	C	0.014	0.003	9.48E-05	-0.012	0.024	-0.008	0.033	-0.017	0.036	-0.034	0.019	-0.014	0.038	-0.013	0.047	0.005	0.012	-0.003	0.016	-0.002	0.037
rs13092376	3	196516288	C	A	0.014	0.003	9.51E-05	-0.017	0.024	-0.011	0.033	-0.028	0.036	-0.015	0.019	0.017	0.036	-0.033	0.045	-0.022	0.012	-0.026	0.015	0.004	0.036
rs115558925	4	69765782	A	C	0.029	0.004	1.55E-04	-0.033	0.042	-0.065	0.057	0.008	0.062	-0.038	0.032	-0.035	0.064	-0.021	0.078	0.009	0.022	0.024	0.029	-0.066	0.065
rs151450	4	88016514	G	A	0.017	0.003	1.45E-04	-0.032	0.024	0.012	0.033	-0.086	0.036	0.013	0.019	0.046	0.035	0.049	0.044	-0.019	0.012	-0.011	0.015	-0.032	0.036
rs1126673	4	100045616	C	T	0.020	0.003	1.66E-04	0.006	0.025	-0.023	0.034	0.046	0.038	0.005	0.019	0.003	0.036	0.049	0.046	0.022	0.013	0.028	0.016	0.002	0.038
rs79800919	4	124787383	G	A	0.026	0.004	1.36E-04	0.000	0.037	0.003	0.049	-0.007	0.054	0.043	0.028	0.004	0.051	0.049	0.065	0.020	0.018	0.028	0.023	0.029	0.054
rs11727331	4	145091680	A	G	0.040	0.006	1.55E-04	-0.048	0.059	-0.029	0.080	-0.062	0.089	-	-	-	-	-	-	-	-	-	-	-	-
rs10003923	4	175086079	C	T	0.015	0.003	1.14E-04	0.031	0.024	0.039	0.032	0.021	0.036	0.009	0.018	-0.086	0.035	0.068	0.044	0.009	0.012	0.002	0.015	0.051	0.037
rs6869704	5	122833667	T	C	0.017	0.003	1.37E-04	0.009	0.024	0.000	0.032	0.017	0.036	0.001	0.018	0.016	0.035	-0.011	0.043	0.004	0.012	0.015	0.015	0.011	0.035
rs274555	5	131722951	C	T	0.018	0.003	1.51E-04	-0.005	0.024	-0.010	0.032	0.003	0.036	0.011	0.019	0.028	0.036	0.004	0.044	0.037	0.012	0.047	0.015	-0.022	0.035
rs12515233	5	158417109	C	A	0.019	0.003	1.05E-04	-0.003	0.031	-0.031	0.042	0.035	0.047	0.020	0.024	-0.013	0.046	-0.068	0.058	-0.007	0.015	0.002	0.020	-0.122	0.046
rs72835688	6	12811955	C	T	0.030	0.005	1.02E-04	-0.033	0.054	-0.081	0.074	0.027	0.080	-	-	-	-	-	-	-	-	-	-	-	-
rs9379764	6	25414023	G	T	0.018	0.003	1.23E-04	-0.001	0.028	0.020	0.038	-0.028	0.042	-0.010	0.022	-0.012	0.042	0.056	0.052	0.015	0.014	-0.002	0.018	0.095	0.041
rs198851	6	26104632	T	G	0.047	0.003	5.59E-04	0.003	0.034	0.002	0.046	0.000	0.050	-0.012	0.025	-0.012	0.049	0.054	0.060	-0.014	0.016	-0.021	0.021	-0.017	0.049
rs200484	6	27775674	A	G	0.023	0.004	1.23E-04	0.014	0.040	0.029	0.053	-0.002	0.060	0.076	0.030	-0.019	0.059	0.049	0.075	-0.112	0.019	-0.135	0.024	0.059	0.060
rs853684	6	28294550	T	C	0.024	0.003	2.84E-04	0.008	0.025	0.015	0.033	0.002	0.037	0.021	0.019	0.017	0.036	0.033	0.046	-0.024	0.012	-0.033	0.015	0.032	0.036
rs9267488	6	31514247	A	G	0.022	0.004	1.22E-04	-0.031	0.038	0.010	0.052	-0.083	0.057	0.108	0.029	0.075	0.059	0.027	0.075	-0.151	0.019	-0.172	0.024	0.057	0.059
rs2395943	6	42940673	A	G	0.020	0.003	1.93E-04	-0.023	0.024	0.013	0.032	-0.067	0.036	0.011	0.018	0.052	0.035	0.020	0.044	0.010	0.012	0.026	0.015	-0.057	0.035
rs12210538	6	110760008	G	A	0.017	0.003	9.99E-05	-0.051	0.028	-0.056	0.038	-0.048	0.042	0.002	0.022	-0.013	0.043	0.067	0.053	-0.003	0.014	-0.010	0.018	0.002	0.042
rs1490384	6	126851160	C	T	0.019	0.003	1.79E-04	0.078	0.024	0.113	0.032	0.036	0.035	0.013	0.018	0.000	0.035	-0.004	0.042	0.000	0.012	0.002	0.015	0.031	0.035
rs11753995	6	160575366	G	A	0.025	0.003	1.71E-04	0.005	0.032	0.002	0.044	0.012	0.048	0.013	0.024	0.059	0.047	0.118	0.059	0.030	0.016	0.034	0.020	0.049	0.048
rs4410790	7	17284577	T	C	0.031	0.003	4.43E-04	-0.016	0.025	-0.060	0.034	0.037	0.038	0.009	0.019	0.043	0.036	0.032	0.044	0.000	0.012	-0.008	0.015	0.012	0.036
rs55696093	7	21605973	G	A	0.026	0.003	2.31E-04	0.001	0.029	0.006	0.040	-0.001	0.044	-0.014	0.022	0.064	0.043	-0.008	0.054	0.029	0.015	0.042	0.019	0.008	0.044
rs57720904	7	28180099	C	T	0.030	0.005	1.36E-04	-0.023	0.040	-0.060	0.054	0.025	0.060	-0.020	0.030	-0.063	0.055	0.082	0.070	-0.015	0.020	-0.008	0.025	-0.056	0.057
rs58699591	7	97876704	C	T	0.023	0.003	1.39E-04	0.049	0.033	0.058	0.044	0.036	0.049	0.010	0.025	-0.020	0.052	0.016	0.062	0.014	0.016	0.008	0.021	0.067	0.049
rs33963055	8	23407178	T	C	0.019	0.003	1.07E-04	-0.063	0.032	-0.041	0.043	-0.088	0.047	0.008	0.024	0.007	0.046	0.019	0.058	0.003	0.016	0.014	0.020	-0.006	0.047
rs34664882	8	41543675	A	G	0.076	0.007	3.85E-04	-0.010	0.072	-0.108	0.098	0.107	0.106	0.105	0.060	-0.131	0.121	0.148	0.146	-0.057	0.037	-0.023	0.048	-0.025	0.110
rs2923430	8	42391195	C	T	0.018	0.003	1.57E-04	-0.033	0.024	-0.069	0.033	0.012	0.037	0.002	0.019	-0.027	0.036	0.038	0.044	0.000	0.012	0.016	0.015	-0.031	0.036
rs8192870	8	59412066	T	G	0.015	0.003	9.94E-05	-0.022	0.025	-0.010	0.034	-0.035	0.037	0.063	0.019	0.065	0.036	0.049	0.045	0.000	0.012	0.000	0.015	-0.031	0.037
rs6986601	8	131026260	G	A	0.014	0.003	9.66E-05	-0.018	0.024	-0.024	0.032	-0.013	0.036	0.026	0.018	0.090	0.035	-0.062	0.043	0.022	0.012	0.025	0.015	0.035	0.036
rs79170239	9	94044272	T	C	0.035	0.003	3.78E-04	0.007	0.030	0.045	0.041	-0.033	0.045	0.002	0.023	-0.012	0.044	-0.041	0.054	-0.012	0.015	0.000	0.019	-0.059	0.045
rs3118753	9	97051962	C	T	0.014	0.003	9.68E-05	0.029	0.025	0.019	0.033	0.042	0.037	-0.025	0.019	-0.001	0.040	-0.068	0.048	0.032	0.012	0.042	0.016	0.014	0.037
rs2519093	9	136141870	C	T	0.024	0.003	1.76E-04	-0.197	0.029	-0.176	0.040	-0.224	0.044	0.031	0.023	0.068	0.043	0.019	0.054	0.038	0.015	0.013	0.019	0.138	0.044
rs34755157	9	137113606	C	T	0.023	0.003	1.52E-04	-0.034	0.031	0.002	0.042	-0.074	0.046	0.015	0.024	0.010	0.046	0.042	0.057	-0.011	0.015	-0.025	0.019	0.026	0.046

Table S1 | Summary statistics for genetic association of total bilirubin levels and risk of ten cancers

					Total bilirubin			Pancreatic cancer overall		Pancreatic cancer in men		Pancreatic cancer in women		Renal cell cancer overall		Renal cell cancer in men		Renal cell cancer in women		Lung cancer overall		Lung cancer overall in ever smokers		Lung cancer overall in never smokers	
SNPs	Chromosome	Base pair position	Effect allele	Reference allele	Beta	SE	Explained variance (%)	Beta	SE	Beta	SE	Beta	SE	Beta	SE	Beta	SE	Beta	SE	Beta	SE	Beta	SE	Beta	SE
rs10761756	10	65172328	C	T	0.040	0.003	7.81E-04	0.036	0.024	0.064	0.032	-0.002	0.035	0.012	0.018	-0.047	0.035	0.021	0.043	0.020	0.012	0.013	0.015	0.015	0.035
rs17476364	10	71094504	C	T	0.091	0.004	1.64E-03	-0.046	0.041	-0.119	0.056	0.043	0.061	-0.022	0.032	0.004	0.066	-0.015	0.081	0.008	0.021	-0.007	0.026	0.015	0.063
rs2901610	10	94536864	C	T	0.022	0.003	2.47E-04	0.023	0.024	0.044	0.032	-0.005	0.035	-0.015	0.018	-0.033	0.035	-0.058	0.044	0.000	0.012	-0.019	0.015	0.030	0.035
rs12768009	10	96525865	G	A	0.024	0.004	1.46E-04	-0.071	0.034	-0.025	0.045	-0.123	0.051	-0.017	0.026	0.001	0.049	-0.046	0.062	-0.005	0.016	0.011	0.021	-0.068	0.049
rs2792751	10	113940329	T	C	0.021	0.003	1.81E-04	0.001	0.026	0.003	0.035	-0.001	0.039	0.018	0.020	-0.007	0.038	-0.030	0.048	-0.005	0.013	-0.006	0.016	-0.043	0.039
rs2245095	10	122857596	C	T	0.048	0.004	3.65E-04	0.017	0.040	0.047	0.054	-0.025	0.062	0.027	0.031	-0.012	0.059	0.070	0.073	-0.009	0.020	-0.022	0.025	0.018	0.061
rs11601507	11	5701074	A	C	0.028	0.005	1.03E-04	0.005	0.046	0.010	0.062	0.002	0.069	-0.016	0.036	-0.105	0.070	0.051	0.083	-0.003	0.024	-0.026	0.030	-0.013	0.071
rs360139	11	9775091	G	A	0.016	0.003	1.21E-04	-	-	-	-	-	-	0.008	0.019	0.035	0.038	-0.038	0.046	-	-	-	-	-	-
rs10832027	11	13357183	G	A	0.024	0.003	2.48E-04	0.017	0.025	0.028	0.034	0.006	0.038	-0.028	0.019	-0.064	0.037	0.075	0.046	-0.008	0.012	-0.037	0.016	0.061	0.037
rs174554	11	61579463	A	G	0.021	0.003	2.02E-04	0.000	0.025	-0.002	0.034	0.001	0.038	0.028	0.019	0.004	0.037	0.044	0.046	0.032	0.012	0.046	0.016	0.023	0.038
rs499974	11	75455021	A	C	0.029	0.003	2.18E-04	0.026	0.031	0.041	0.041	0.005	0.046	0.007	0.024	0.051	0.044	-0.031	0.056	0.015	0.015	-0.001	0.019	0.080	0.045
rs717662	11	100493995	T	C	0.026	0.004	1.34E-04	-0.002	0.037	-0.018	0.050	0.010	0.055	-0.024	0.029	-0.063	0.054	-0.073	0.068	-0.004	0.018	-0.016	0.023	-0.022	0.054
rs3741298	11	116657561	C	T	0.032	0.003	3.11E-04	-0.046	0.029	-0.064	0.040	-0.027	0.043	-0.017	0.023	-0.013	0.044	0.037	0.054	0.004	0.014	-0.010	0.018	0.033	0.043
rs76895963	12	4384844	G	T	0.062	0.010	1.54E-04	-	-	-	-	-	-	-	-	-	-	-	-	-0.044	0.051	0.029	0.064	-0.327	0.146
rs73080739	12	20761863	G	A	0.033	0.004	1.91E-04	0.033	0.040	-0.015	0.054	0.090	0.058	-0.002	0.030	0.052	0.057	0.031	0.073	0.011	0.020	0.025	0.025	0.017	0.060
rs4149056	12	21331549	C	T	0.183	0.003	8.57E-03	-0.072	0.032	-0.095	0.043	-0.045	0.049	-0.054	0.024	-0.113	0.045	-0.033	0.056	-0.014	0.016	-0.027	0.020	-0.028	0.047
rs1283809	12	21980618	C	T	0.021	0.003	1.16E-04	0.001	0.032	0.039	0.043	-0.046	0.047	-0.027	0.025	0.086	0.049	-0.022	0.061	0.025	0.016	0.032	0.020	0.043	0.046
rs36124182	12	24214934	A	G	0.049	0.007	1.68E-04	0.034	0.058	-0.012	0.080	0.085	0.084	-0.065	0.045	-0.020	0.088	0.069	0.115	0.022	0.029	0.002	0.037	-0.028	0.083
rs4760682	12	48512285	C	A	0.017	0.003	9.22E-05	-0.016	0.031	-0.024	0.042	-0.009	0.046	0.007	0.024	0.072	0.048	-0.016	0.059	-0.028	0.016	-0.024	0.020	-0.070	0.048
rs10876376	12	53261822	A	G	0.016	0.003	1.31E-04	-0.099	0.024	-0.104	0.032	-0.095	0.035	0.021	0.018	0.043	0.035	-0.025	0.044	-0.007	0.012	0.002	0.015	-0.036	0.035
rs2657879	12	56865338	G	A	0.018	0.003	9.46E-05	-0.040	0.030	-0.018	0.041	-0.064	0.045	-0.012	0.024	-0.043	0.047	-0.044	0.058	0.019	0.015	0.009	0.020	0.068	0.045
rs3184504	12	111884608	T	C	0.038	0.003	7.25E-04	-0.031	0.024	-0.023	0.032	-0.041	0.035	-0.029	0.018	-0.021	0.035	-0.081	0.044	-0.034	0.012	-0.025	0.015	-0.044	0.035
rs12811045	12	115527503	A	G	0.017	0.003	9.89E-05	0.012	0.030	0.022	0.040	-0.005	0.045	-0.010	0.023	-0.019	0.047	-0.065	0.057	0.012	0.015	0.002	0.019	-0.008	0.044
rs7135337	12	121404155	C	A	0.016	0.003	1.20E-04	-0.102	0.024	-0.109	0.032	-0.092	0.036	-0.042	0.018	-0.039	0.035	-0.065	0.044	-0.019	0.012	-0.013	0.015	0.010	0.035
rs139763750	14	51049397	A	G	0.038	0.006	1.45E-04	0.026	0.056	0.053	0.076	-0.002	0.082	0.078	0.040	0.065	0.077	0.146	0.093	-	-	-	-	-	-
rs61984409	14	64730021	A	C	0.015	0.003	9.73E-05	-0.011	0.025	-0.026	0.033	0.007	0.036	0.010	0.019	-0.021	0.037	0.000	0.045	-	-	-	-	-	-
rs339969	15	60883281	C	A	0.026	0.003	3.20E-04	0.004	0.024	0.007	0.033	0.004	0.036	0.009	0.019	0.023	0.037	0.007	0.045	-0.013	0.012	-0.022	0.015	0.019	0.036
rs17184256	15	63785421	G	A	0.022	0.003	2.14E-04	0.041	0.025	0.030	0.035	0.051	0.038	0.023	0.020	0.046	0.037	-0.047	0.047	-0.025	0.013	-0.031	0.016	0.013	0.038
rs67257650	15	73979507	T	C	0.017	0.003	1.42E-04	-0.024	0.024	-0.031	0.033	-0.012	0.036	0.001	0.019	0.032	0.035	-0.018	0.045	0.016	0.012	0.006	0.015	0.018	0.036
rs181207	16	28513530	C	T	0.020	0.003	1.76E-04	0.002	0.026	0.014	0.034	-0.013	0.039	-0.035	0.020	-0.013	0.040	-0.107	0.050	-0.028	0.013	-0.033	0.017	0.020	0.040
rs4575545	16	79755446	G	A	0.017	0.003	1.22E-04	-0.008	0.026	-0.007	0.035	-0.008	0.039	-0.052	0.020	-0.098	0.038	-0.177	0.047	-0.013	0.013	-0.030	0.016	0.025	0.038
rs7185774	16	81249927	C	T	0.015	0.003	9.57E-05	0.003	0.025	0.045	0.034	-0.046	0.037	-0.019	0.019	-0.038	0.036	-0.075	0.045	0.005	0.012	0.007	0.015	-0.063	0.036
rs247827	16	84582825	G	A	0.019	0.003	1.10E-04	0.013	0.031	0.018	0.042	0.005	0.046	0.068	0.024	0.068	0.048	0.041	0.059	0.008	0.016	0.011	0.020	0.035	0.047
rs2968478	16	88858646	T	G	0.023	0.003	2.60E-04	-0.037	0.025	-0.013	0.033	-0.064	0.037	-0.031	0.019	-0.033	0.036	-0.081	0.045	0.001	0.012	0.006	0.016	0.004	0.037
rs7222046	17	7806529	G	A	0.017	0.003	1.34E-04	-0.019	0.024	-0.008	0.032	-0.034	0.036	-0.026	0.018	-0.007	0.035	0.005	0.043	-0.006	0.012	0.016	0.015	-0.037	0.035
rs1045599	17	15879910	C	T	0.016	0.003	1.24E-04	0.018	0.024	0.018	0.032	0.015	0.036	0.007	0.018	-0.004	0.035	0.019	0.044	0.000	0.012	-0.007	0.015	0.039	0.036
rs7213285	17	27206029	A	G	0.020	0.003	1.07E-04	0.080	0.032	0.068	0.044	0.099	0.049	0.005	0.025	-0.011	0.048	-0.007	0.058	-0.010	0.016	-0.024	0.020	0.074	0.047
rs1477141	17	53361838	G	T	0.014	0.003	1.03E-04	0.003	0.024	0.002	0.032	0.004	0.035	-0.047	0.018	-0.059	0.034	-0.112	0.044	0.003	0.012	-0.006	0.015	0.031	0.038
rs764424	17	57505604	G	A	0.015	0.003	1.12E-04	-0.016	0.024	-0.053	0.032	0.027	0.035	0.023	0.018	-0.006	0.034	0.055	0.043	0.013	0.012	0.020	0.015	0.001	0.035
rs11079420	17	59253051	A	G	0.020	0.003	1.33E-04	0.093	0.029	0.098	0.039	0.087	0.043	0.009	0.022	-0.014	0.043	0.039	0.054	-0.028	0.014	-0.029	0.018	-0.057	0.041
rs7223257	17	61787684	T	C	0.017	0.003	1.33E-04	-0.045	0.025	-0.061	0.034	-0.028	0.037	0.031	0.019	0.079	0.037	0.048	0.046	0.017	0.012	0.015	0.016	-0.004	0.038
rs12601919	17	65825374	A	G	0.021	0.003	1.35E-04	-0.053	0.030	-0.057	0.040	-0.048	0.045	-0.003	0.024	0.027	0.046	-0.061	0.057	-0.049	0.015	-0.045	0.019	-0.113	0.044
rs2909210	17	66456481	T	G	0.015	0.003	1.17E-04	-0.030	0.024	-0.076	0.032	0.021	0.035	0.024	0.018	0.036	0.035	0.039	0.044	-0.009	0.012	-0.013	0.015	0.007	0.035
rs1135688	17	73827205	C	T	0.015	0.003	9.06E-05	0.042	0.026	0.055	0.035	0.024	0.038	0.043	0.020	0.046	0.038	0.081	0.048	-0.021	0.013	-0.033	0.016	-0.037	0.038
rs379149	17	76126586	C	T	0.027	0.004	1.54E-04	0.037	0.039	-0.002	0.052	0.088	0.058	-0.039	0.034	-	-	-	-	-0.050	0.024	-0.060	0.031	-0.038	0.069
rs112896133	19	12429782	G	A	0.078	0.011	1.55E-04	0.047	0.130	0.253	0.170	-0.262	0.205	0.043	0.099	-	-	-	-	-0.048	0.059	-0.073	0.074	0.224	0.170
rs12609744	19	12994140	T	C	0.016	0.003	1.10E-04	-0.013	0.026	-0.008	0.035	-0.018	0.039	-0.016	0.020	-0.047	0.038	0.010	0.046	-0.007	0.013	0.006	0.016	-0.034	0.039
rs59616136	19	17252041	A	G	0.036	0.004	2.15E-04	-0.030	0.044	-0.036	0.060	-0.021	0.065	0.002	0.035	0.021	0.073	-0.145	0.087	-0.011	0.021	-0.010	0.027	-0.027	0.061
rs58542926	19	19379549	T	C	0.041	0.005	2.34E-04	0.064	0.045	0.104	0.059	0.007	0.068	0.052	0.035	0.081	0.066	0.100	0.084	-0.014	0.023	-0.019	0.029	0.014	0.069
rs157595	19	45425460	G	A	0.017	0.003	1.31E-04	0.004	0.025	0.033	0.034	-0.031	0.038	-0.028	0.020	-0.027	0.039	-0.008	0.048	0.008	0.013	0.003	0.016	0.026	0.037
rs676388	19	49211969	C	T	0.021	0.003	2.29E-04	0.023	0.024	-0.008	0.032	0.063	0.036	-0.026	0.018	0.000	0.035	-0.007	0.043	0.025	0.012	0.009	0.015	0.087	0.035
rs12459419	19	51728477	T	C	0.015	0.003	9.91E-05	0.051	0.026	0.040	0.035	0.065	0.038	-0.006	0.020	0.002	0.039	0.044	0.048	-0.017	0.013	-0.036	0.016	0.022	0.038
rs117113213	20	39165692	G	A	0.046	0.007	1.32E-04	-0.107	0.068	-0.181	0.094	-0.027	0.100	-	-	-	-	-	-	-	-	-	-	-	-
rs6129760	20	39746403	G	A	0.018	0.003	1.37E-04	0.015	0.026	0.045	0.035	-0.018	0.038	0.012	0.019	0.001	0.037	-0.036	0.046	-0.015	0.013	-0.019	0.016	-0.015	0.037
rs1800961	20	43042364	T	C	0.062	0.007	2.38E-04	-0.144	0.070	-0.045	0.095	-0.264	0.106	0.015	0.054	-0.064	0.110	0.026	0.131	-0.075	0.034	-0.040	0.043	-0.020	0.111
rs4820091	22	21940189	T	G	0.018	0.003	9.78E-05	-0.010	0.031	-0.006	0.041	-0.018	0.047	-0.008	0.023	-0.008	0.045	0.019	0.057	0.021	0.015	0.036	0.019	0.024	0.046
rs855791	22	37462936	G	A	0.028	0.003	3.94E-04	-0.020	0.024	-0.021	0.032	-0.018	0.036	0.006	0.018	-0.024	0.035	-0.054	0.044	-0.029	0.012	-0.027	0.015	-0.080	0.035
rs738408	22	44324730	T	C	0.019	0.003	1.19E-04	-0.006	0.029	-0.021	0.038	0.013	0.043	0.031	0.022	-0.013	0.041	0.016	0.051	-0.011	0.014	-0.005	0.017	-0.054	0.042

Table S1 | Summary statistics for genetic association of total bilirubin levels and risk of ten cancers

					Lung adenocarcinoma		Squamous cell lung cancer		Small cell Lung cancer		Ovarian cancer overall		Ovarian serous cancer		Breast cancer overall		Breast ER positive cancer		Breast ER negative cancer		Endometrial cancer		Prostate cancer		Melanoma		Neuroblastoma	
SNPs	Chromosome	Base pair position	Effect allele	Reference allele	Beta	SE	Beta	SE	Beta	SE	Beta	SE	Beta	SE	Beta	SE	Beta	SE	Beta	SE	Beta	SE	Beta	SE	Beta	SE	Beta	SE
rs2375279	1	25541931	C	T	0.003	0.023	-0.013	0.027	0.011	0.042	0.005	0.017	0.001	0.019	-0.005	0.010	-0.003	0.011	0.011	0.017	-0.007	0.023	-0.007	0.012	0.082	0.078	-0.089	0.060
rs17513135	1	40035686	C	T	0.008	0.019	-0.005	0.022	-0.027	0.034	0.034	0.014	0.038	0.016	-0.014	0.008	-0.012	0.009	-0.024	0.014	-0.035	0.019	-0.020	0.010	-0.008	0.065	-	-
rs6682423	1	63171063	T	C	-0.021	0.017	-0.043	0.020	0.050	0.031	0.016	0.013	0.016	0.014	-0.003	0.007	-0.005	0.008	0.007	0.012	-	-	-0.010	0.009	-	-	0.075	0.045
rs1762486	1	107627697	A	G	0.000	0.017	-0.013	0.020	0.021	0.032	0.024	0.013	0.024	0.014	0.000	0.007	0.001	0.008	-0.018	0.012	0.009	0.017	-0.004	0.009	-	-	-	-
rs61812598	1	154420087	A	G	0.013	0.017	-0.008	0.019	-0.011	0.030	0.014	0.012	0.012	0.013	-0.007	0.006	-0.012	0.008	0.001	0.012	0.031	0.016	0.016	0.008	-0.019	0.057	0.003	0.044
rs857725	1	158607935	T	G	-0.013	0.018	-0.016	0.021	-0.055	0.033	-0.002	0.013	0.005	0.015	-0.003	0.007	0.004	0.009	-0.042	0.013	-	-	-0.021	0.009	-0.004	0.062	-0.058	0.049
rs788644	1	202256962	G	T	-0.026	0.016	-0.006	0.019	0.012	0.029	-	-	-	-	0.001	0.006	0.013	0.008	-0.031	0.012	0.004	0.016	-0.014	0.008	-0.055	0.057	-0.101	0.043
rs1874121	1	220969049	T	C	-0.006	0.018	0.010	0.021	0.011	0.033	-	-	-	-	-0.012	0.007	-0.018	0.009	-0.013	0.013	-0.025	0.018	0.001	0.009	-	-	-	-
rs556107	1	234853059	T	C	0.024	0.016	-0.023	0.019	0.002	0.030	-0.029	0.012	-0.016	0.013	0.004	0.007	0.009	0.008	-0.003	0.012	0.006	0.016	0.019	0.008	0.097	0.055	-0.026	0.043
rs13030095	2	26026598	A	G	0.000	0.018	0.026	0.021	0.038	0.033	0.003	0.013	-0.011	0.015	-0.004	0.007	-0.002	0.009	-0.011	0.013	-0.010	0.017	0.012	0.009	-0.044	0.061	0.023	0.046
rs2053799	2	32883197	G	A	0.028	0.017	0.018	0.019	0.004	0.030	0.005	0.012	-0.007	0.013	0.013	0.006	0.010	0.008	0.021	0.012	-0.020	0.016	-0.010	0.008	-	-	-	-
rs4671605	2	64887382	T	C	0.013	0.018	0.019	0.021	0.053	0.034	-0.013	0.014	-0.017	0.015	0.019	0.007	0.023	0.009	0.022	0.013	0.006	0.018	0.006	0.010	-0.003	0.063	0.010	0.048
rs6734238	2	113841030	A	G	-0.006	0.017	-0.031	0.019	-0.041	0.030	0.019	0.012	0.021	0.013	0.005	0.006	0.005	0.008	0.011	0.012	-0.038	0.016	-0.022	0.008	0.083	0.057	-0.017	0.044
rs1047891	2	211540507	A	C	0.016	0.018	-0.007	0.021	-0.043	0.033	0.010	0.013	0.009	0.014	0.011	0.007	0.001	0.008	0.036	0.013	-0.025	0.018	0.012	0.009	-	-	-	-
rs6731997	2	232560411	G	A	-0.001	0.020	-0.001	0.023	-0.002	0.036	0.005	0.015	0.003	0.016	-0.014	0.008	-0.016	0.009	0.001	0.014	-0.032	0.020	0.010	0.010	0.001	0.069	0.059	0.053
rs4973588	2	233834975	A	G	0.011	0.022	0.017	0.026	-0.024	0.041	-0.010	0.016	-0.002	0.018	-0.001	0.009	-0.004	0.011	0.016	0.016	0.031	0.022	0.008	0.012	-	-	-0.091	0.061
**rs6431625**	**2**	**234637912**	**C**	**T**	**-0.004**	**0.017**	**-0.018**	**0.019**	**-0.031**	**0.031**	**-0.024**	**0.012**	**-0.037**	**0.013**	**0.001**	**0.006**	**0.008**	**0.008**	**-0.020**	**0.012**	**-0.006**	**0.016**	**0.000**	**0.008**	**0.048**	**0.058**	**0.029**	**0.044**
rs10929023	2	235169902	G	A	-0.011	0.018	-0.001	0.021	0.050	0.033	0.004	0.013	0.005	0.015	0.003	0.007	0.005	0.009	0.007	0.014	-0.027	0.018	0.004	0.010	0.084	0.058	0.044	0.047
rs2267846	3	48556339	A	G	0.011	0.018	0.019	0.021	0.040	0.034	-0.033	0.014	-0.031	0.015	0.009	0.007	0.006	0.009	0.018	0.013	0.010	0.018	0.033	0.009	-0.153	0.062	0.009	0.048
rs9826148	3	114464858	C	T	-0.014	0.025	-0.018	0.030	0.025	0.047	0.023	0.019	0.046	0.021	0.007	0.010	0.009	0.012	-0.011	0.018	-0.022	0.025	0.010	0.013	0.049	0.090	-	-
rs6779903	3	135720851	T	G	0.036	0.018	0.003	0.021	0.029	0.033	-0.016	0.013	-0.019	0.015	0.006	0.007	0.005	0.008	0.013	0.013	0.001	0.018	-0.004	0.009	-0.002	0.061	0.040	0.048
rs1052618	3	136574501	A	G	0.019	0.017	0.005	0.020	0.022	0.032	0.010	0.013	0.006	0.014	0.013	0.007	0.015	0.008	0.025	0.012	-0.029	0.017	-0.018	0.009	-0.052	0.059	0.017	0.047
rs1482852	3	156798294	A	G	-0.004	0.017	0.010	0.019	-0.032	0.031	0.033	0.012	0.047	0.013	0.006	0.006	0.005	0.008	0.012	0.012	-0.017	0.016	-0.022	0.008	-	-	-0.029	0.044
rs61791066	3	170713380	C	T	-0.005	0.023	-0.017	0.027	-0.015	0.043	-0.037	0.017	-0.036	0.019	0.016	0.009	0.007	0.011	0.028	0.016	-0.003	0.023	-0.024	0.012	0.034	0.082	0.031	0.063
rs11917973	3	195838613	T	C	0.014	0.017	0.016	0.020	-0.026	0.031	0.003	0.012	0.011	0.014	0.015	0.007	0.018	0.008	0.014	0.012	-0.020	0.017	-0.001	0.009	-	-	-	-
rs13092376	3	196516283	C	A	-0.023	0.017	-0.026	0.019	-0.007	0.030	0.017	0.012	0.019	0.014	-0.004	0.007	-0.003	0.008	-0.004	0.012	-0.035	0.016	0.004	0.008	-	-	-	-
rs115558925	4	69765782	A	C	0.005	0.030	-0.021	0.036	-0.010	0.057	0.007	0.021	-0.017	0.023	-0.018	0.012	-0.018	0.014	-0.025	0.021	0.017	0.029	0.018	0.015	-	-	-	-
rs151450	4	88016514	G	A	-0.006	0.016	-0.037	0.019	-0.017	0.030	0.009	0.012	0.007	0.013	-0.013	0.006	-0.016	0.008	-0.009	0.012	0.022	0.016	-0.012	0.008	-	-	-	-
rs1126673	4	100045616	C	T	0.000	0.017	0.031	0.020	0.011	0.032	-0.017	0.013	-0.019	0.014	-0.012	0.007	-0.017	0.008	0.007	0.012	-0.016	0.017	-0.004	0.009	0.052	0.059	0.034	0.046
rs79800919	4	124787383	G	A	0.053	0.025	0.033	0.029	-0.018	0.045	0.002	0.018	-0.001	0.020	0.013	0.010	0.019	0.012	0.011	0.018	-0.027	0.025	0.027	0.013	0.044	0.086	-	-
rs11727331	4	145091680	A	G	-	-	-	-	-	-	0.008	0.027	0.012	0.030	-	-	-	-	-	-	0.020	0.037	0.023	0.018	-	-	-	-
rs10003923	4	175086079	C	T	0.025	0.017	0.000	0.020	0.029	0.031	0.006	0.013	0.006	0.014	0.007	0.007	0.013	0.008	-0.006	0.012	-0.004	0.017	-0.001	0.009	-0.050	0.055	-	-
rs6869704	5	122833667	T	C	0.009	0.016	0.021	0.019	-0.050	0.030	0.005	0.012	0.007	0.013	0.005	0.006	0.006	0.007	0.011	0.011	-0.026	0.016	0.007	0.008	0.100	0.055	-0.009	0.043
rs274555	5	131722951	C	T	0.027	0.016	0.035	0.019	0.063	0.030	0.012	0.012	0.021	0.013	0.024	0.006	0.026	0.007	-0.003	0.012	-0.009	0.016	-	-	-0.039	0.056	-	-
rs12515233	5	158417109	C	A	-0.030	0.021	0.002	0.025	0.046	0.039	-0.020	0.015	-0.028	0.017	0.044	0.008	0.046	0.010	0.051	0.015	0.033	0.021	0.005	0.010	0.102	0.074	-	-
rs72835688	6	12811955	C	T	-	-	-	-	-	-	0.004	0.029	0.012	0.032	-0.016	0.016	-0.020	0.019	-0.004	0.029	0.054	0.040	0.017	0.020	-	-	-	-
rs9379764	6	25414023	G	T	0.049	0.019	-0.010	0.022	-0.014	0.035	-0.013	0.014	-0.017	0.015	0.009	0.007	0.009	0.009	0.001	0.014	-0.001	0.019	0.013	0.010	-	-	-	-
rs198851	6	26104632	T	G	-0.025	0.023	-0.014	0.026	0.000	0.041	-0.001	0.016	0.004	0.018	0.000	0.009	-0.002	0.011	-0.012	0.016	0.052	0.022	-0.033	0.011	-0.070	0.075	-0.036	0.062
rs200484	6	27775674	A	G	-0.040	0.027	-0.188	0.031	-0.130	0.049	0.011	0.019	-0.001	0.021	0.045	0.011	0.051	0.013	0.036	0.020	-	-	0.042	0.013	-0.139	0.132	0.045	0.069
rs853684	6	28294550	T	C	-0.001	0.017	-0.058	0.019	-0.026	0.031	0.036	0.012	0.033	0.014	-0.009	0.007	-0.006	0.008	-0.021	0.012	0.012	0.016	0.006	0.008	-0.042	0.067	0.067	0.044
rs9267488	6	31514247	A	G	-0.091	0.027	-0.230	0.030	-0.198	0.048	-0.019	0.019	-0.028	0.021	0.025	0.010	0.027	0.013	-0.002	0.019	0.109	0.025	0.055	0.013	-	-	-	-
rs2395943	6	42940673	A	G	0.014	0.016	-0.003	0.019	0.006	0.030	0.004	0.012	0.006	0.013	0.011	0.006	0.021	0.008	-0.005	0.011	-0.027	0.016	-0.028	0.008	-0.008	0.057	-0.025	0.044
rs12210538	6	110760008	G	A	-0.003	0.019	-0.005	0.022	-0.007	0.036	0.029	0.014	0.020	0.015	-0.008	0.007	-0.005	0.009	-0.004	0.013	0.027	0.019	0.012	0.010	0.117	0.065	-	-
rs1490384	6	126851160	C	T	-0.009	0.016	0.009	0.019	0.016	0.029	0.001	0.012	0.005	0.013	0.002	0.006	0.000	0.008	0.018	0.012	0.000	0.016	-0.012	0.008	-0.019	0.056	0.031	0.043
rs11753995	6	160575366	G	A	0.028	0.022	0.015	0.025	0.011	0.040	-0.010	0.016	0.004	0.018	0.004	0.008	0.001	0.010	0.016	0.015	0.027	0.021	0.037	0.011	0.047	0.073	-0.002	0.058
rs4410790	7	17284577	T	C	-0.003	0.017	-0.020	0.019	-0.033	0.031	0.000	0.012	0.006	0.014	0.004	0.006	0.009	0.008	-0.006	0.012	-0.016	0.016	0.004	0.008	-0.054	0.057	-0.014	0.044
rs55696093	7	21605973	G	A	0.009	0.020	0.045	0.023	0.025	0.037	-0.024	0.015	-0.035	0.017	0.007	0.008	0.017	0.010	-0.004	0.015	0.001	0.020	0.010	0.010	-	-	0.046	0.052
rs57720904	7	28180099	C	T	-0.044	0.027	0.018	0.031	0.001	0.050	-0.053	0.019	-0.052	0.022	0.028	0.011	0.029	0.012	0.019	0.019	-0.046	0.027	0.013	0.014	-	-	-0.063	0.075
rs58699591	7	97876704	C	T	0.012	0.023	-0.017	0.026	0.094	0.042	0.011	0.016	0.016	0.018	-0.008	0.009	-0.016	0.010	0.014	0.016	-0.033	0.021	-0.066	0.011	-	-	-	-
rs33963055	8	23407178	T	C	0.009	0.021	-0.009	0.025	-0.011	0.039	0.003	0.016	0.008	0.017	-0.013	0.008	-0.010	0.010	-0.020	0.015	0.035	0.021	0.001	0.010	-0.083	0.073	-	-
rs34664882	8	41543675	A	G	-0.121	0.052	-0.038	0.060	-0.053	0.095	0.047	0.037	0.073	0.041	-0.001	0.020	-0.030	0.025	0.015	0.037	0.060	0.048	0.006	0.026	0.115	0.163	-	-
rs2923430	8	42391195	C	T	-0.001	0.017	0.013	0.019	0.001	0.030	0.024	0.012	0.025	0.014	0.008	0.007	0.010	0.009	0.011	0.013	-0.017	0.017	-0.002	0.009	0.005	0.056	0.025	0.044
rs8192870	8	59412066	T	G	-0.009	0.017	-0.004	0.020	0.000	0.031	-0.002	0.012	-0.010	0.014	0.016	0.007	0.014	0.008	0.003	0.012	0.020	0.017	-0.001	0.008	0.052	0.060	-	-
rs6986601	8	131026260	G	A	0.016	0.016	0.028	0.019	0.010	0.030	-0.002	0.012	0.002	0.013	0.007	0.006	0.012	0.007	-0.001	0.011	-0.005	0.016	-0.004	0.008	-	-	-	-
rs79170239	9	94044272	T	C	-0.020	0.021	0.027	0.024	-0.015	0.038	-0.012	0.015	-0.016	0.017	-0.008	0.008	-0.006	0.010	0.006	0.015	0.009	0.020	0.032	0.010	-0.056	0.073	-0.053	0.056
rs3118753	9	97051962	C	T	0.031	0.017	0.056	0.020	-0.045	0.031	0.029	0.012	0.039	0.014	-0.016	0.007	-0.017	0.008	-0.027	0.012	-0.006	0.016	-0.007	0.008	0.092	0.057	-0.033	0.045
rs2519093	9	136141870	C	T	0.059	0.021	0.038	0.024	-0.005	0.037	-0.092	0.015	-0.109	0.016	-0.043	0.008	-0.042	0.009	-0.035	0.014	-0.056	0.020	-0.006	0.010	-0.019	0.071	-	-
rs34755157	9	137113606	C	T	-0.013	0.021	0.006	0.024	-0.019	0.038	-0.009	0.015	-0.015	0.017	0.007	0.008	0.009	0.010	-0.003	0.015	0.009	0.021	-0.009	0.011	-	-	-	-

Table S1 | Summary statistics for genetic association of total bilirubin levels and risk of ten cancers

					Lung adenocarcinoma		Squamous cell lung cancer		Small cell Lung cancer		Ovarian cancer overall		Ovarian serous cancer		Breast cancer overall		Breast ER positive cancer		Breast ER negative cancer		Endometrial cancer		Prostate cancer		Melanoma		Neuroblastoma	
SNPs	Chromosome	Base pair position	Effect allele	Reference allele	Beta	SE	Beta	SE	Beta	SE	Beta	SE	Beta	SE	Beta	SE	Beta	SE	Beta	SE	Beta	SE	Beta	SE	Beta	SE	Beta	SE
rs10761756	10	65172328	C	T	0.009	0.016	0.020	0.019	0.038	0.030	-0.007	0.012	-0.003	0.013	0.021	0.006	0.028	0.008	0.002	0.012	0.012	0.016	0.000	0.008	-0.022	0.055	0.055	0.043
rs17476364	10	71094504	C	T	0.004	0.028	-0.001	0.033	-0.013	0.053	-0.016	0.021	-0.025	0.023	0.009	0.011	0.012	0.013	0.018	0.020	-0.035	0.027	0.011	0.014	-	-	-	-
rs2901610	10	94536864	C	T	-0.004	0.016	0.007	0.019	0.005	0.030	-0.009	0.012	-0.007	0.013	-0.008	0.006	-0.014	0.008	-0.005	0.012	0.008	0.016	0.005	0.008	-	-	-0.053	0.043
rs12768009	10	96525865	G	A	-0.001	0.023	0.003	0.026	0.063	0.042	0.039	0.017	0.040	0.019	0.002	0.009	-0.004	0.010	0.016	0.016	0.001	0.022	-0.022	0.011	0.005	0.078	-0.126	0.062
rs2792751	10	113940329	T	C	-0.005	0.018	-0.007	0.020	0.007	0.032	0.010	0.013	0.016	0.014	-0.013	0.007	-0.015	0.008	-0.008	0.013	0.012	0.018	0.015	0.009	0.016	0.061	0.074	0.047
rs2245095	10	122857596	C	T	0.003	0.028	0.020	0.032	0.001	0.050	0.023	0.020	0.035	0.023	0.032	0.011	0.030	0.013	0.001	0.019	-0.005	0.027	-0.003	0.014	0.037	0.095	-0.070	0.075
rs11601507	11	5701074	A	C	0.021	0.033	-0.037	0.039	0.018	0.061	-0.065	0.025	-0.074	0.028	0.003	0.013	0.018	0.016	0.016	0.024	0.016	0.034	-0.068	0.017	-0.145	0.107	-	-
rs360139	11	9775091	G	A	-	-	-	-	-	-	-0.006	0.012	0.000	0.014	-0.021	0.007	-0.021	0.008	-0.031	0.012	-0.004	0.017	-0.019	0.009	0.039	0.060	0.004	0.045
rs10832027	11	13357183	G	A	-0.008	0.017	-0.026	0.020	0.012	0.031	-0.026	0.012	-0.025	0.014	0.002	0.007	0.000	0.008	0.011	0.012	-0.003	0.017	-0.014	0.009	0.042	0.060	0.052	0.046
rs174554	11	61579463	A	G	0.035	0.017	0.039	0.020	0.026	0.031	-0.014	0.012	-0.008	0.014	0.008	0.007	0.000	0.008	0.031	0.012	-0.021	0.017	0.002	0.009	0.029	0.059	-0.010	0.048
rs499974	11	75455021	A	C	0.032	0.021	-0.022	0.024	0.013	0.038	0.030	0.015	0.027	0.017	-0.012	0.008	-0.027	0.010	0.042	0.015	-0.031	0.021	0.000	0.010	0.002	0.074	0.088	0.057
rs717662	11	100493995	T	C	-0.022	0.025	0.010	0.029	-0.059	0.047	0.016	0.019	0.006	0.021	-0.014	0.010	-0.037	0.012	0.040	0.018	0.032	0.025	0.016	0.013	-	-	-0.149	0.066
rs3741298	11	116657561	C	T	0.006	0.020	0.030	0.023	-0.072	0.036	0.002	0.015	0.007	0.016	-0.017	0.008	-0.027	0.009	-0.013	0.014	-0.001	0.020	-0.009	0.010	-0.086	0.067	-	-
rs76895963	12	4384844	G	T	-0.072	0.068	-0.021	0.081	0.094	0.120	0.016	0.044	0.032	0.049	0.001	0.026	-0.005	0.030	0.015	0.048	-0.131	0.065	0.038	0.032	-	-	-	-
rs73080739	12	20761863	G	A	-0.009	0.027	0.025	0.032	0.074	0.050	0.019	0.020	0.027	0.022	-0.004	0.010	-0.017	0.012	0.015	0.019	-0.021	0.026	-	-	-0.043	0.098	-	-
rs4149056	12	21331549	C	T	-0.020	0.022	0.002	0.025	-0.057	0.040	0.032	0.016	0.036	0.018	0.010	0.008	0.012	0.010	0.013	0.015	0.079	0.021	-0.004	0.011	-0.019	0.076	-	-
rs1283809	12	21980618	C	T	0.035	0.022	0.037	0.026	0.002	0.041	0.038	0.016	0.028	0.018	-0.002	0.009	0.004	0.010	-0.012	0.016	-	-	-	-	-0.037	0.073	-	-
rs36124182	12	24214934	A	G	0.025	0.039	-0.042	0.046	0.044	0.075	0.042	0.030	0.033	0.033	0.014	0.017	0.017	0.020	0.015	0.030	0.003	0.042	-	-	-	-	-	-
rs4760682	12	48512285	C	A	-0.014	0.022	-0.051	0.026	-0.018	0.041	0.004	0.016	-0.001	0.018	-0.006	0.008	-0.008	0.010	-	-	-0.014	0.021	-	-	-	-	-	-
rs10876376	12	53261822	A	G	-0.003	0.016	0.001	0.019	0.004	0.030	-0.003	0.012	0.008	0.013	0.005	0.006	-0.005	0.007	0.021	0.011	0.002	0.016	-0.019	0.008	-0.092	0.056	0.028	0.043
rs2657879	12	56865338	G	A	0.020	0.021	-0.002	0.025	-0.013	0.039	-0.034	0.015	-0.038	0.017	0.006	0.009	0.001	0.010	0.031	0.016	0.024	0.021	-	-	-0.024	0.072	-	-
rs3184504	12	111884608	T	C	-0.034	0.016	-0.034	0.019	-0.053	0.030	-0.007	0.012	-0.002	0.013	-0.031	0.006	-0.030	0.007	-0.033	0.011	-0.099	0.016	-0.019	0.008	0.047	0.055	0.060	0.043
rs12811045	12	115527503	A	G	0.000	0.021	0.015	0.024	-0.021	0.038	0.002	0.014	0.010	0.016	0.002	0.008	-0.004	0.009	0.017	0.014	0.006	0.020	0.008	0.010	0.086	0.069	-	-
rs7135337	12	121404155	C	A	-0.023	0.016	0.019	0.019	-0.026	0.030	-0.065	0.012	-0.080	0.013	0.017	0.006	0.009	0.008	0.032	0.011	0.001	0.016	-0.013	0.008	0.084	0.057	-0.061	0.044
rs139763750	14	51049397	A	G	-	-	-	-	-	-	0.013	0.027	0.002	0.031	-0.022	0.015	-0.035	0.018	-0.042	0.027	-0.031	0.036	-0.001	0.020	-	-	-	-
rs61984409	14	64730021	A	C	-	-	-	-	-	-	0.020	0.012	0.009	0.014	-0.014	0.006	-0.014	0.008	-0.023	0.012	-0.003	0.016	-0.023	0.008	-	-	-	-
rs339969	15	60883281	C	A	0.008	0.017	-0.034	0.019	0.004	0.031	0.035	0.012	0.045	0.014	-0.010	0.007	-0.007	0.008	-0.013	0.012	-0.011	0.016	-0.006	0.008	-0.051	0.057	0.024	0.044
rs17184256	15	63785421	G	A	-0.015	0.017	-0.049	0.020	-0.102	0.032	0.007	0.013	-0.005	0.014	0.004	0.007	0.008	0.008	-0.017	0.012	0.050	0.017	0.006	0.009	-	-	-	-
rs67257650	15	73979507	T	C	0.019	0.016	0.036	0.019	0.033	0.030	-0.006	0.012	-0.004	0.013	-0.003	0.006	0.002	0.008	-0.009	0.012	0.020	0.016	0.008	0.008	0.099	0.056	0.034	0.043
rs181207	16	28513530	C	T	-0.008	0.018	-0.043	0.021	-0.053	0.034	-0.005	0.013	0.013	0.015	-0.002	0.007	-0.010	0.009	0.008	0.013	-0.034	0.018	-0.003	0.009	-0.083	0.059	-	-
rs4575545	16	79755446	G	A	-0.028	0.018	-0.012	0.021	0.026	0.032	0.016	0.013	0.015	0.014	0.001	0.007	-0.002	0.008	0.016	0.012	-0.019	0.017	0.007	0.009	0.110	0.059	-	-
rs7185774	16	81249927	C	T	-0.014	0.017	0.020	0.019	0.004	0.031	-0.004	0.012	-0.012	0.014	-0.002	0.007	-0.001	0.008	0.000	0.012	0.011	0.017	0.000	0.009	-0.079	0.059	-0.100	0.045
rs247827	16	84582825	G	A	0.004	0.022	0.023	0.025	0.059	0.040	-0.014	0.016	-0.017	0.017	0.009	0.008	0.003	0.010	0.018	0.015	0.023	0.021	-0.024	0.011	-	-	-	-
rs2968478	16	88858646	T	G	0.001	0.017	0.023	0.020	-0.039	0.031	0.016	0.012	0.015	0.014	-0.009	0.007	-0.007	0.008	-0.013	0.012	-0.003	0.017	-0.009	0.009	-	-	-	-
rs7222046	17	7806529	G	A	-0.016	0.016	0.024	0.019	-0.002	0.030	0.010	0.012	0.008	0.014	0.019	0.007	0.021	0.008	0.020	0.012	-0.008	0.017	0.030	0.009	0.000	0.056	0.042	0.044
rs1045599	17	15879910	C	T	-0.003	0.016	-0.030	0.019	-0.032	0.030	-0.009	0.012	-0.018	0.014	-0.003	0.006	-0.006	0.008	0.004	0.012	-0.024	0.016	-0.007	0.008	0.002	0.056	-0.017	0.044
rs7213285	17	27206029	A	G	-0.019	0.022	0.008	0.026	-0.012	0.041	-0.015	0.016	-0.017	0.018	0.007	0.009	0.001	0.010	0.009	0.016	0.002	0.022	0.007	0.011	-	-	-	-
rs1477141	17	53361838	G	T	0.017	0.017	-0.019	0.019	-0.011	0.031	0.011	0.013	0.025	0.014	0.010	0.007	0.005	0.008	0.013	0.012	0.028	0.017	0.006	0.009	0.013	0.055	-	-
rs764424	17	57505604	G	A	0.024	0.016	0.000	0.019	-0.010	0.030	-0.007	0.012	-0.009	0.013	0.008	0.006	0.013	0.007	0.002	0.011	0.000	0.016	0.013	0.008	-0.077	0.056	-0.015	0.043
rs11079420	17	59253051	A	G	-0.037	0.019	-0.034	0.023	-0.041	0.035	0.029	0.014	0.041	0.016	0.003	0.008	0.012	0.009	-0.017	0.014	-0.016	0.019	0.023	0.010	-0.006	0.067	0.046	0.053
rs7223257	17	61787684	T	C	0.037	0.017	-0.022	0.020	0.071	0.032	-0.008	0.012	-0.008	0.014	0.009	0.007	0.004	0.008	0.001	0.012	-0.016	0.017	-0.024	0.008	-0.066	0.060	-0.053	0.046
rs12601919	17	65825374	A	G	-0.052	0.021	-0.042	0.024	-0.152	0.037	0.023	0.015	0.034	0.016	-0.002	0.008	0.002	0.009	-0.025	0.014	-0.088	0.020	0.027	0.010	-0.055	0.068	0.046	0.054
rs2909210	17	66456481	T	G	-0.013	0.016	-0.027	0.019	0.020	0.030	0.014	0.012	0.026	0.013	0.004	0.006	-0.005	0.008	0.025	0.012	-0.015	0.016	0.013	0.008	-	-	-	-
rs1135688	17	73827205	C	T	-0.024	0.017	-0.025	0.020	0.013	0.032	-0.013	0.013	-0.014	0.015	0.015	0.007	0.020	0.009	0.017	0.013	0.027	0.018	-0.004	0.009	-0.022	0.058	-	-
rs379149	17	76126586	C	T	-0.028	0.032	-0.084	0.038	-0.052	0.061	-	-	-	-	-	-	-	-	-	-	-0.033	0.029	-0.029	0.015	-	-	-	-
rs112896133	19	12429782	G	A	0.008	0.081	-0.099	0.091	-0.192	0.144	-0.118	0.054	-0.155	0.060	-0.048	0.031	-0.058	0.037	-0.002	0.057	-0.178	0.072	-	-	-	-	-	-
rs12609744	19	12994140	T	C	-0.023	0.018	0.031	0.021	0.017	0.033	0.022	0.013	0.015	0.015	0.009	0.007	0.013	0.008	-0.012	0.013	-0.030	0.018	0.020	0.009	0.025	0.060	-	-
rs59616136	19	17252041	A	G	-0.002	0.028	-0.027	0.034	-0.098	0.054	-0.013	0.020	-0.020	0.022	0.011	0.011	0.012	0.013	-0.008	0.019	0.017	0.027	0.002	0.014	-	-	-	-
rs58542926	19	19379549	T	C	-0.045	0.031	0.006	0.036	-0.042	0.058	0.018	0.022	0.016	0.025	0.018	0.012	0.025	0.014	0.027	0.021	0.019	0.029	0.006	0.015	0.098	0.101	-	-
rs157595	19	45425460	G	A	0.017	0.017	-0.001	0.020	-0.027	0.032	0.013	0.012	0.007	0.014	-0.014	0.007	-0.011	0.008	-0.005	0.012	-0.007	0.017	-0.005	0.009	-	-	-	-
rs676388	19	49211969	C	T	0.068	0.016	-0.029	0.019	0.056	0.030	0.022	0.012	0.000	0.013	0.012	0.006	0.015	0.008	-0.005	0.012	0.004	0.016	0.009	0.008	0.028	0.055	-0.113	0.043
rs12459419	19	51728477	T	C	-0.014	0.018	-0.017	0.020	-0.029	0.032	-0.010	0.013	-0.017	0.014	0.008	0.007	0.010	0.008	0.012	0.012	-0.006	0.017	0.012	0.009	-	-	-	-
rs117113213	20	39165692	G	A	-	-	-	-	-	-	-	-	-	-	-	-	-	-	-	-	-	-	-0.057	0.025	-	-	-	-
rs6129760	20	39746403	G	A	-0.015	0.017	0.002	0.020	-0.011	0.032	0.005	0.012	-0.006	0.014	-0.007	0.007	-0.007	0.008	0.000	0.012	-0.024	0.017	0.005	0.009	-0.056	0.061	-0.088	0.046
rs1800961	20	43042364	T	C	-0.048	0.047	-0.058	0.055	-0.125	0.089	-0.003	0.034	0.013	0.038	-0.033	0.018	-0.027	0.021	-0.057	0.033	0.060	0.044	-	-	0.036	0.169	-	-
rs4820091	22	21940189	T	G	0.043	0.021	-0.012	0.024	0.034	0.038	-0.007	0.015	0.003	0.017	-0.007	0.008	-0.009	0.009	0.007	0.014	0.014	0.020	0.004	0.010	0.003	0.070	-0.064	0.055
rs855791	22	37462936	G	A	-0.027	0.016	-0.028	0.019	-0.017	0.030	0.021	0.012	0.020	0.013	-0.003	0.006	0.001	0.008	-0.013	0.012	-0.007	0.016	-0.004	0.008	0.030	0.055	-0.044	0.043
rs738408	22	44324730	T	C	0.006	0.019	-0.042	0.022	-0.011	0.035	-0.011	0.014	-0.007	0.016	-0.004	0.008	-0.002	0.009	-0.021	0.014	0.000	0.020	-0.022	0.010	-	-	-	-

Table S2 | Results of sensitivity Mendelian randomization methods for total bilirubin levels and risk of ten cancers

				Likelihood-based method					Inverse-variance weighted method				Weigthed median approach				Modal-based method				MR-Egger Simex approach				MR-Egger intercept approach				MR-PRESSO approach		
**Cancer outcome**	**Subtype**	**SNP set**	**n SNP**	**OR**	**LCI**	**UCI**	***P***	***Phet***	**OR**	**LCI**	**UCI**	***P***	**OR**	**LCI**	**UCI**	***P***	**OR**	**LCI**	**UCI**	***P***	**OR**	**LCI**	**UCI**	***P***	**Est**	**LCI**	**UCI**	***P***	***P* Global**	***P* Distortion**	**Outlier SNPs**
**Pancreatic cancer**	overall	*UGT1A1* SNP	1	1.02	0.95	1.11	0.56																								
		Non-*UGT1A1* SNPs	113	0.74	0.61	0.89	1.7E-03	3.0E-08	0.74	0.61	0.90	2.E-03	0.68	0.49	0.95	0.02	0.69	0.51	0.94	0.02	0.69	0.43	1.10	0.12	0.002	-0.007	0.011	0.63	<1e-04	0.28	rs10876376,rs2519093
		Pleiotropy out SNPs	91	0.85	0.66	1.11	0.23	2.2E-03	0.85	0.66	1.11	0.23	0.96	0.63	1.45	0.84	1.02	0.50	2.09	0.95	1.05	0.41	2.664	0.92	-0.005	-0.021	0.010	0.51	2.1E-03	0.23	rs10876376,rs7135337
	men	*UGT1A1* SNP	1	1.06	0.95	1.18	0.27																								
		Non-*UGT1A1* SNPs	113	0.75	0.58	0.96	0.02	2.5E-03	0.75	0.58	0.97	0.03	0.60	0.39	0.93	0.02	0.60	0.39	0.92	0.02	0.60	0.35	1.05	0.07	0.007	-0.005	0.019	0.27	2.2E-03	0.72	rs2519093
		Pleiotropy out SNPs	91	0.89	0.63	1.27	0.53	0.11	0.89	0.63	1.26	0.51	0.96	0.56	1.64	0.89	1.18	0.26	5.26	0.83	0.89	0.29	2.73	0.83	6.9E-06	-0.021	0.021	1.00	0.10	-	-
	women	*UGT1A1* SNP	1	0.98	0.87	1.10	0.71																								
		Non-*UGT1A1* SNPs	113	0.73	0.55	0.97	0.03	0.01	0.73	0.55	0.97	0.03	0.78	0.49	1.26	0.32	0.81	0.51	1.29	0.38	0.81	0.44	1.49	0.49	-0.004	-0.017	0.010	0.60	0.01	0.57	rs2519093
		Pleiotropy out SNPs	91	0.81	0.55	1.20	0.29	0.11	0.81	0.55	1.19	0.29	0.96	0.52	1.76	0.90	0.93	0.36	2.42	0.89	1.44	0.42	4.97	0.56	-0.013	-0.036	0.011	0.29	0.11	-	-
**Renal cell cancer**	overall	*UGT1A1* SNP	1	1.01	0.95	1.07	0.78																								
		Non-*UGT1A1* SNPs	111	0.95	0.82	1.09	0.45	1.2E-03	0.95	0.82	1.09	0.45	0.76	0.61	0.96	0.02	0.77	0.61	0.97	0.03	0.78	0.57	1.06	0.11	0.007	-4.E-04	0.014	0.06	8.0E-04	-	-
		Pleiotropy out SNPs	89	1.09	0.89	1.33	0.43	3.9E-04	1.08	0.89	1.32	0.43	1.05	0.77	1.43	0.78	1.14	0.70	1.87	0.60	1.09	0.51	2.34	0.82	4.2E-04	-0.012	0.013	0.95	2.0E-04	-	-
	men	*UGT1A1* SNP	1	1.01	0.89	1.13	0.93																								
		Non-*UGT1A1* SNPs	109	0.76	0.57	1.00	0.05	0.41	0.76	0.57	1.00	0.05	0.56	0.35	0.89	0.01	0.56	0.37	0.86	0.01	0.50	0.30	0.82	0.01	0.014	5.E-04	0.027	0.04	0.37	-	-
		Pleiotropy out SNPs	87	0.88	0.59	1.30	0.50	0.34	0.88	0.59	1.29	0.51	0.74	0.41	1.34	0.33	0.65	0.23	1.82	0.41	0.39	0.11	1.35	0.14	0.017	-0.007	0.041	0.17	0.35	-	-
	women	*UGT1A1* SNP	1	1.10	0.95	1.28	0.20																								
		Non-*UGT1A1* SNPs	109	1.05	0.74	1.48	0.80	0.41	1.05	0.74	1.48	0.80	0.84	0.48	1.48	0.56	0.93	0.54	1.61	0.80	1.03	0.55	1.92	0.93	0.001	-0.016	0.018	0.90	0.42	-	-
		Pleiotropy out SNPs	87	1.06	0.65	1.72	0.83	0.39	1.06	0.65	1.71	0.83	1.32	0.64	2.70	0.46	1.84	0.57	5.87	0.31	2.21	0.48	10.17	0.31	-0.015	-0.045	0.015	0.31	0.41	-	-
**Lung cancer**	overall	*UGT1A1* SNP	1	0.98	0.94	1.02	0.31																								
		Non-*UGT1A1* SNPs	109	0.91	0.83	1.00	0.06	1.2E-15	0.91	0.83	1.00	0.06	0.93	0.79	1.09	0.36	0.93	0.80	1.09	0.39	0.84	0.64	1.10	0.20	0.002	-0.002	0.007	0.29	<1e-04	0.25	rs200484,rs9267488
		Pleiotropy out SNPs	87	0.92	0.81	1.05	0.22	1.3E-14	0.92	0.81	1.05	0.23	1.01	0.81	1.26	0.95	1.06	0.73	1.52	0.77	0.78	0.42	1.48	0.45	0.003	-0.005	0.011	0.43	<1e-04	0.05	rs200484,rs9267488
	ever smokers	*UGT1A1* SNP	1	0.95	0.91	1.00	0.07																								
		Non-*UGT1A1* SNPs	109	0.86	0.76	0.96	0.01	2.3E-10	0.86	0.76	0.97	0.01	0.86	0.71	1.06	0.16	0.85	0.71	1.03	0.09	0.79	0.58	1.07	0.13	0.003	-0.003	0.009	0.35	<1e-04	0.28	rs200484,rs9267488
		Pleiotropy out SNPs	87	0.89	0.75	1.05	0.16	2.4E-10	0.89	0.76	1.05	0.17	0.93	0.71	1.23	0.64	0.98	0.66	1.44	0.91	0.72	0.34	1.50	0.38	0.004	-0.006	0.015	0.38	<1e-04	0.15	rs200484,rs9267488
	never smokers	*UGT1A1* SNP	1	0.99	0.88	1.12	0.91																								
		Non-*UGT1A1* SNPs	109	1.01	0.76	1.34	0.95	0.03	1.01	0.76	1.34	0.95	0.86	0.53	1.40	0.56	0.94	0.60	1.48	0.80	0.83	0.47	1.47	0.53	0.006	-0.007	0.020	0.37	0.03	-	-
		Pleiotropy out SNPs	87	0.97	0.66	1.44	0.88	0.10	0.97	0.66	1.43	0.88	1.18	0.63	2.23	0.61	1.28	0.48	3.42	0.62	1.03	0.29	3.72	0.96	-0.001	-0.025	0.022	0.91	0.10	-	-
	adenocarcinoma	*UGT1A1* SNP	1	0.99	0.94	1.05	0.81																								
		Non-*UGT1A1* SNPs	109	0.96	0.84	1.09	0.54	6.6E-04	0.96	0.84	1.09	0.54	0.90	0.72	1.13	0.38	0.91	0.74	1.11	0.35	0.80	0.60	1.06	0.12	0.006	-3.E-04	0.012	0.06	9.0E-04	0.78	rs676388
		Pleiotropy out SNPs	87	1.02	0.85	1.22	0.86	1.7E-03	1.02	0.85	1.22	0.87	1.03	0.77	1.37	0.87	1.02	0.68	1.52	0.94	0.76	0.39	1.46	0.41	0.006	-0.005	0.017	0.26	1.5E-03	0.15	rs676388
	squamous cell	*UGT1A1* SNP	1	0.97	0.91	1.03	0.35																								
		Non-*UGT1A1* SNPs	109	0.85	0.73	0.99	0.04	5.8E-11	0.85	0.73	0.99	0.04	1.01	0.78	1.30	0.95	0.99	0.77	1.26	0.91	0.93	0.62	1.38	0.71	-0.003	-0.010	0.004	0.44	<1e-04	0.25	rs200484,rs9267488
		Pleiotropy out SNPs	87	0.80	0.65	0.99	0.04	4.5E-12	0.81	0.66	0.99	0.05	0.99	0.70	1.42	0.97	1.08	0.65	1.80	0.77	0.74	0.28	1.95	0.54	0.002	-0.011	0.014	0.78	<1e-04	0.17	rs200484,rs9267488
	small cell	*UGT1A1* SNP	1	0.95	0.86	1.05	0.32																								
		Non-*UGT1A1* SNPs	109	0.77	0.61	0.97	0.03	0.02	0.77	0.61	0.98	0.03	0.75	0.50	1.13	0.17	0.77	0.53	1.14	0.20	0.65	0.40	1.06	0.09	0.005	-0.007	0.016	0.40	0.02	0.78	rs9267488
		Pleiotropy out SNPs	87	0.95	0.68	1.33	0.76	3.8E-03	0.95	0.68	1.32	0.76	1.03	0.61	1.73	0.93	1.00	0.43	2.31	0.99	0.72	0.22	2.38	0.59	0.006	-0.014	0.026	0.55	4.0E-03	0.18	rs9267488
**Ovarian cancer**	overall	*UGT1A1* SNP	1	0.96	0.92	1.00	0.05																								
		Non-*UGT1A1* SNPs	111	1.10	1.00	1.21	0.04	6.2E-13	1.10	1.00	1.21	0.04	1.19	1.01	1.40	0.04	1.13	0.97	1.32	0.11	1.11	0.86	1.43	0.43	0.000	-0.005	0.005	1.00	<1e-04	0.56	rs2519093,rs7135337
		Pleiotropy out SNPs	89	1.12	0.98	1.27	0.10	1.9E-06	1.11	0.98	1.27	0.11	1.14	0.93	1.41	0.21	0.91	0.66	1.26	0.57	0.93	0.55	1.57	0.78	0.004	-0.004	0.012	0.30	<1e-04	0.75	rs7135337
	serous	*UGT1A1* SNP	1	0.94	0.90	0.98	5.8E-03																								
		Non-*UGT1A1* SNPs	111	1.12	1.01	1.24	0.04	9.8E-15	1.12	1.01	1.24	0.04	1.22	1.01	1.47	0.04	1.19	1.01	1.41	0.05	1.14	0.85	1.53	0.37	-0.001	-0.006	0.004	0.79	<1e-04	0.53	rs2519093,rs7135337
		Pleiotropy out SNPs	89	1.10	0.95	1.27	0.23	2.9E-08	1.09	0.95	1.26	0.24	1.07	0.84	1.36	0.62	0.96	0.65	1.41	0.84	0.95	0.52	1.73	0.86	0.003	-0.005	0.012	0.44	<1e-04	0.74	rs7135337
**Breast cancer**	overall	*UGT1A1* SNP	1	1.00	0.98	1.02	0.84																								
		Non-*UGT1A1* SNPs	112	1.05	1.00	1.10	0.07	8.5E-23	1.05	1.00	1.10	0.07	1.06	0.97	1.15	0.21	1.06	0.97	1.15	0.19	1.01	0.86	1.18	0.91	0.001	-0.001	0.004	0.31	<1e-04	0.63	rs12515233,rs200484, rs2519093,rs3184504
		Pleiotropy out SNPs	90	1.12	1.04	1.20	1.7E-03	3.2E-15	1.12	1.04	1.20	0.00	1.09	0.95	1.25	0.21	1.06	0.83	1.35	0.63	1.09	0.78	1.52	0.62	0.001	-0.003	0.005	0.64	<1e-04	0.48	rs12515233,rs200484
	ER positive	*UGT1A1* SNP	1	1.01	0.99	1.04	0.29																								
		Non-*UGT1A1* SNPs	112	1.05	0.99	1.11	0.14	1.8E-19	1.05	0.98	1.11	0.15	1.06	0.96	1.18	0.23	1.07	0.97	1.18	0.17	1.02	0.85	1.22	0.84	0.001	-0.002	0.004	0.54	<1e-04	0.72	rs12515233,rs200484, rs2519093,rs3184504
		Pleiotropy out SNPs	91	1.12	1.03	1.22	0.01	4.7E-15	1.12	1.03	1.21	0.01	1.13	0.96	1.33	0.13	1.11	0.83	1.48	0.49	1.06	0.71	1.60	0.77	0.001	-0.004	0.006	0.58	<1e-04	0.77	rs12515233
	ER negative	*UGT1A1* SNP	1	0.97	0.93	1.00	0.08																								
		Non-*UGT1A1* SNPs	112	1.05	0.96	1.16	0.26	1.2E-06	1.05	0.96	1.15	0.27	1.07	0.92	1.25	0.40	1.06	0.92	1.23	0.43	1.00	0.81	1.25	0.97	0.002	-0.003	0.006	0.47	<1e-04	-	-
		Pleiotropy out SNPs	89	1.08	0.95	1.22	0.26	2.1E-04	1.07	0.95	1.22	0.27	1.11	0.90	1.37	0.33	1.13	0.83	1.54	0.45	1.09	0.67	1.76	0.74	-2.2E-05	-0.008	0.008	1.00	<1e-04	-	-
**Endometrial cancer**		*UGT1A1* SNP	1	0.99	0.94	1.04	0.71																								
		Non-*UGT1A1* SNPs	110	0.97	0.86	1.11	0.69	3.7E-11	0.97	0.86	1.11	0.69	1.39	1.14	1.71	2.E-03	1.42	1.15	1.76	2.E-03	1.37	0.99	1.89	0.06	-0.011	-0.017	-0.005	4.E-04	<1e-04	0.46	rs12601919,rs3184504, rs4149056,rs9267488
		Pleiotropy out SNPs	88	0.84	0.70	1.01	0.06	4.0E-04	0.84	0.71	1.01	0.06	0.89	0.67	1.19	0.45	1.02	0.63	1.65	0.94	0.87	0.45	1.65	0.66	-0.001	-0.012	0.009	0.81	5.0E-04	0.98	rs9267488
**Prostate cancer**		*UGT1A1* SNP	1	1.00	0.97	1.03	1.00																								
		Non-*UGT1A1* SNPs	107	0.97	0.91	1.03	0.32	4.7E-24	0.97	0.91	1.03	0.32	0.98	0.88	1.09	0.75	1.00	0.90	1.11	0.98	0.97	0.79	1.19	0.76	-4.0E-05	-0.003	0.003	0.98	<1e-04	0.53	rs11601507,rs58699591, rs9267488
		Pleiotropy out SNPs	87	1.00	0.91	1.10	0.98	1.4E-19	1.00	0.91	1.09	0.98	1.06	0.91	1.23	0.50	1.11	0.89	1.38	0.36	1.09	0.68	1.73	0.73	-0.002	-0.007	0.004	0.47	<1e-04	0.16	rs2267846,rs58699591
**Hodgkin's lymphoma**		*UGT1A1* SNP	1	1.00	0.85	1.19	0.97																								
		Non-*UGT1A1* SNPs	91	0.64	0.42	0.99	0.04	0.19	0.64	0.42	0.99	0.04	0.69	0.35	1.36	0.29	0.67	0.34	1.33	0.26	0.65	0.28	1.47	0.29	-0.001	-0.021	0.020	0.96	0.21	-	-
		Pleiotropy out SNPs	71	0.61	0.32	1.14	0.12	0.15	0.61	0.33	1.14	0.12	0.42	0.16	1.06	0.07	0.27	0.03	2.55	0.26	0.54	0.04	7.29	0.64	0.001	-0.042	0.044	0.97	0.14	-	-
**Melanoma**		*UGT1A1* SNP	1	1.08	0.90	1.31	0.41																								
		Non-*UGT1A1* SNPs	75	1.00	0.60	1.65	1.00	0.67	1.00	0.60	1.66	1.00	0.90	0.41	1.97	0.81	0.89	0.42	1.91	0.77	0.86	0.37	2.00	0.73	0.005	-0.019	0.030	0.68	0.69	-	-
		Pleiotropy out SNPs	56	0.85	0.39	1.87	0.69	0.57	0.85	0.39	1.86	0.69	0.88	0.28	2.76	0.84	0.83	0.14	4.92	0.84	0.79	0.05	12.79	0.87	0.001	-0.052	0.055	0.96	0.59	-	-
**Neuroblastoma**		*UGT1A1* SNP	1	1.05	0.91	1.21	0.51																								
		Non-*UGT1A1* SNPs	57	0.81	0.47	1.40	0.45	0.07	0.82	0.47	1.41	0.46	0.96	0.42	2.21	0.93	3.82	0.72	20.30	0.12	1.52	0.14	16.54	0.72	-0.015	-0.057	0.027	0.49	0.06	-	-
		Pleiotropy out SNPs	45	0.76	0.40	1.45	0.41	0.02	0.75	0.40	1.43	0.39	1.77	0.67	4.72	0.26	4.58	0.65	32.45	0.13	1.41	0.06	34.13	0.83	-0.013	-0.063	0.037	0.60	0.02	-	-

OR: Odds ratio. LCI: Lower confidence interval. UCI: Upper confidence interval. *P*: *P* value. *Phet*: *P* value for SNPs heterogeneity

Figure S1. Scatter plots depicting the genetic associations between bilirubin levels and risk of pancreatic and renal cell cancers, overall and sex subgroups

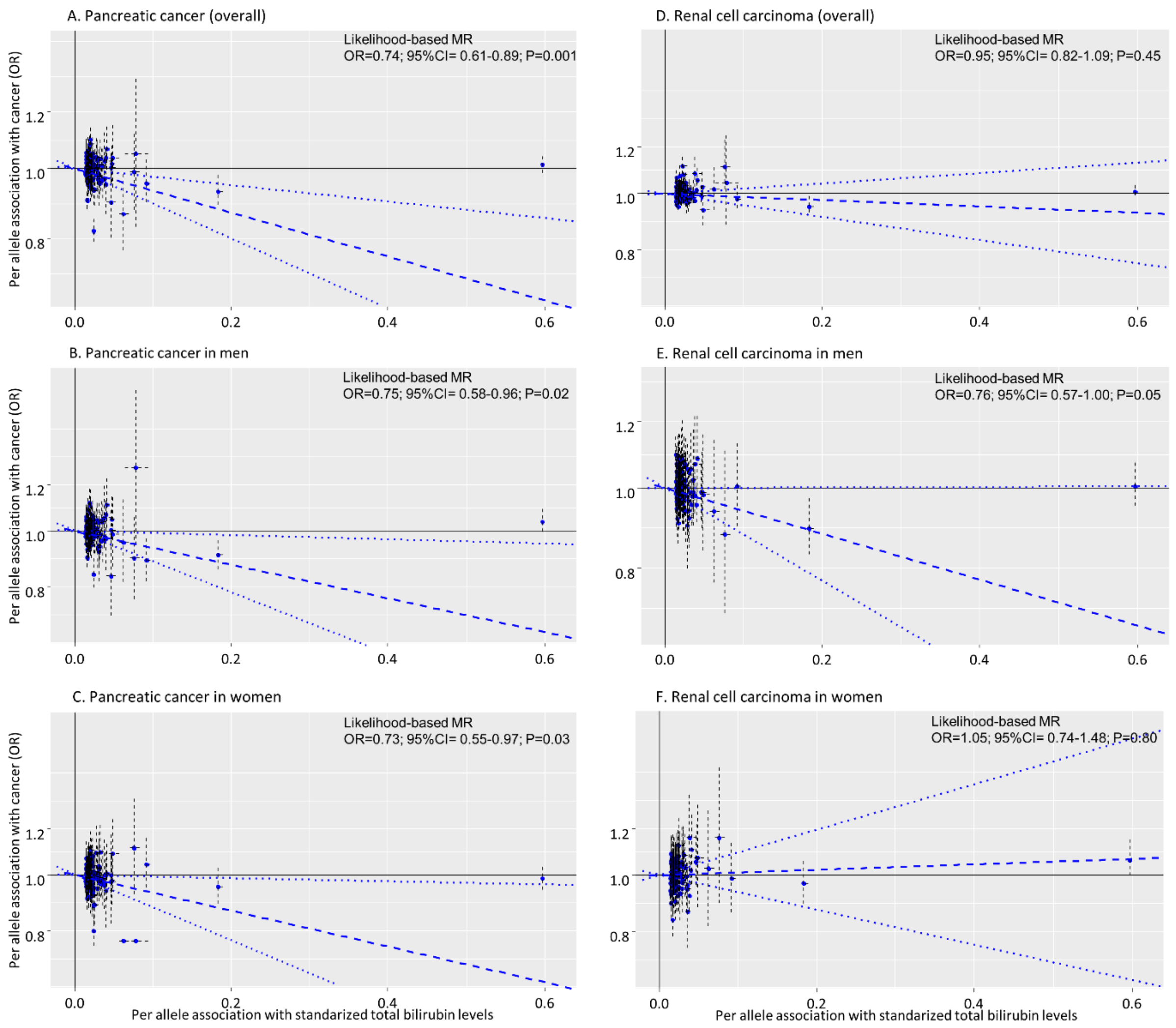

Per allele association of bilirubin SNPs with inverse-normal-transformed bilirubin levels (x axis) and risk of pancreatic cancer and renal cell carcinoma (y axis; logarithmic scale); pancreatic cancer overall (A), pancreatic cancer in men (B), pancreatic cancer in women (C) renal cell carcinoma overall (D), renal cell carcinoma in men (E), renal cell carcinoma in women (F), including the likelihood-based MR estimate (dashed-blue line) and its 95% confidence interval (dotted-blue lines).

Figure S2. Scatter plots depicting the genetic associations between bilirubin levels and lung cancer risk, overall, smoking subgroups, and histological subtypes

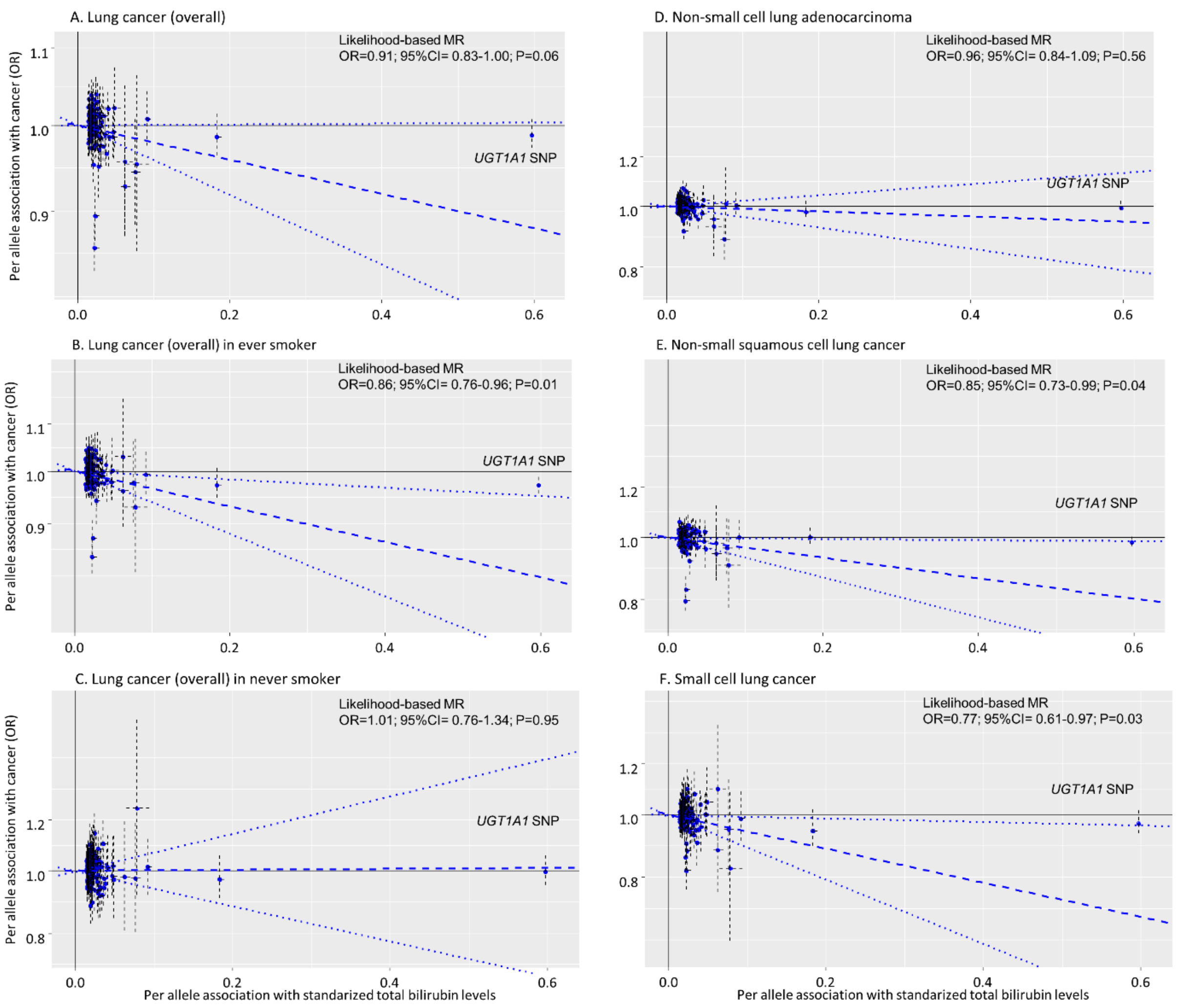

Per allele associations of bilirubin SNPs with inverse-normal-transformed bilirubin levels (x axis) and lung cancer risk (y axis; logarithmic scale); overall (A), in ever smoker (B), in never smoker (C), adenocarcinoma (D), squamous cell lung cancer (E), small cell lung cancer (F), including the likelihood-based MR estimate (dashed-blue line) and its 95% confidence interval (dotted-blue lines).

Figure S3. Scatter plots depicting the genetic associations between bilirubin levels and risk of ovarian, endometrial, and breast cancers, overall and subtypes

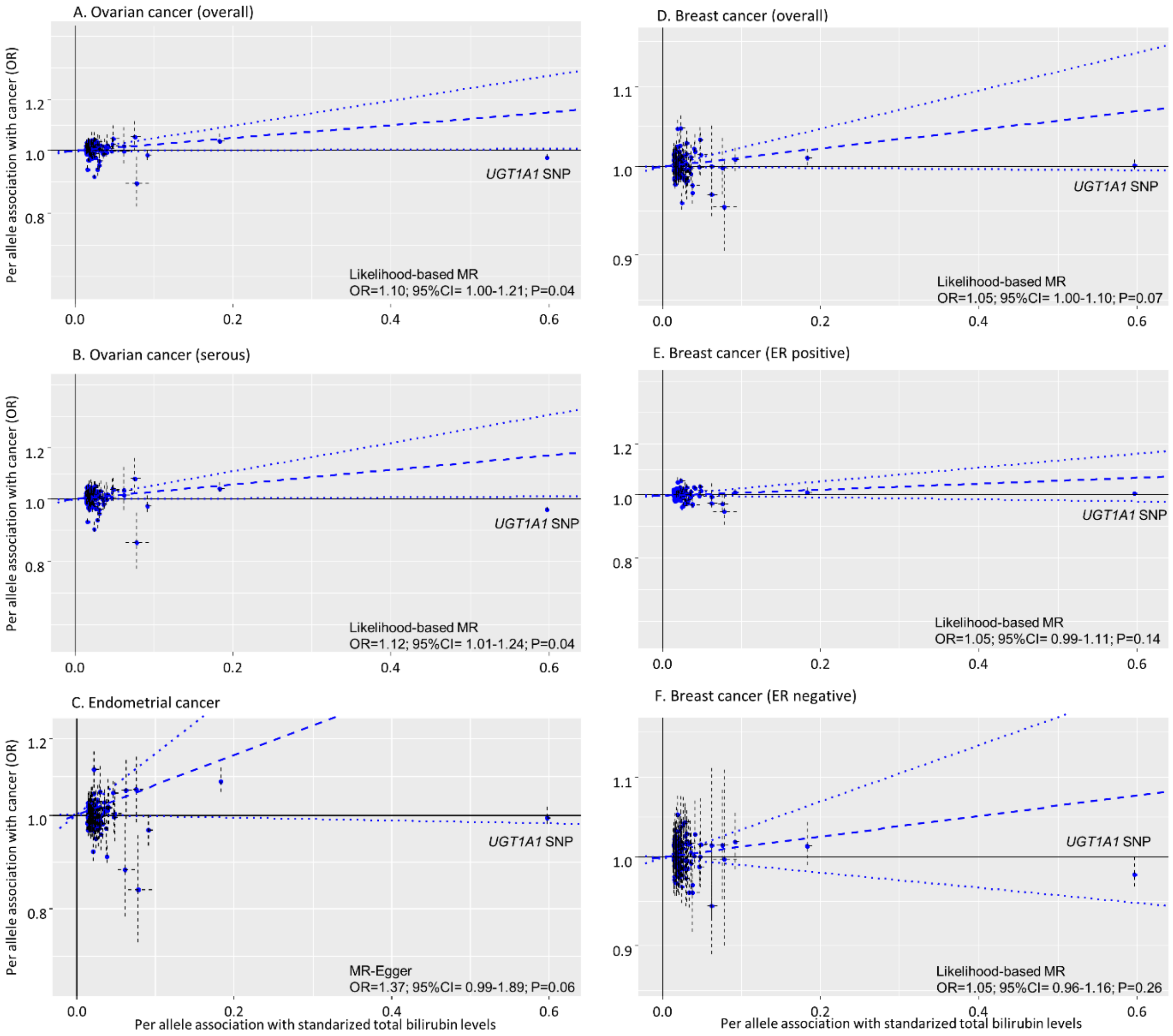

Per allele associations of bilirubin SNPs with inverse-normal-transformed bilirubin levels (x axis) and risk of ovarian, endometrial, and breast cancers (y axis; logarithmic scale); ovarian cancer overall (A), serous ovarian cancer (B), endometrial cancer (C), breast cancer overall (D), ER positive breast cancer (E), ER negative breast cancer (F), including the likelihood-based MR estimate (dashed-blue line) and its 95% confidence interval (dotted-blue lines), with the exception of endometrial cancer, which results are provided by the Egger-MR test.

Figure S4. Scatter plots depicting the genetic association between bilirubin levels and prostate cancer risk

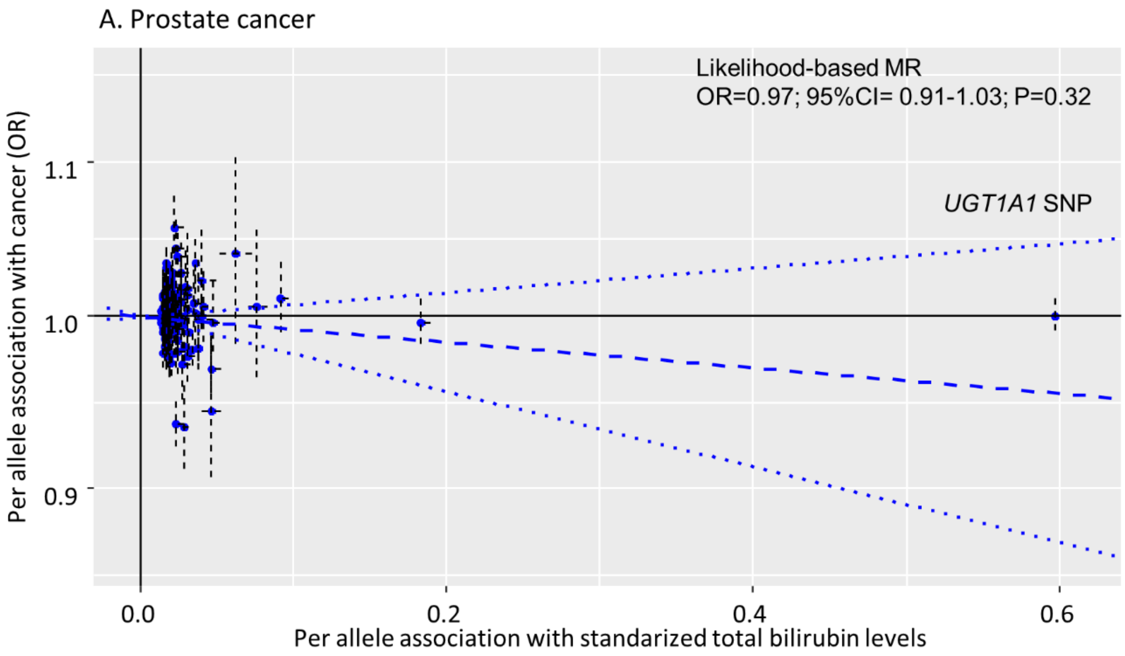

Per allele association of bilirubin SNPs with inverse-normal-transformed bilirubin levels (x axis) and prostate cancer risk (y axis; logarithmic scale), including the likelihood-based MR estimate (dashed-blue line) and its 95% confidence interval (dotted-blue lines).

Figure S5. Scatter plots depicting the genetic associations between bilirubin levels and risk of Hodgkin's lymphoma, melanoma, and neuroblastoma

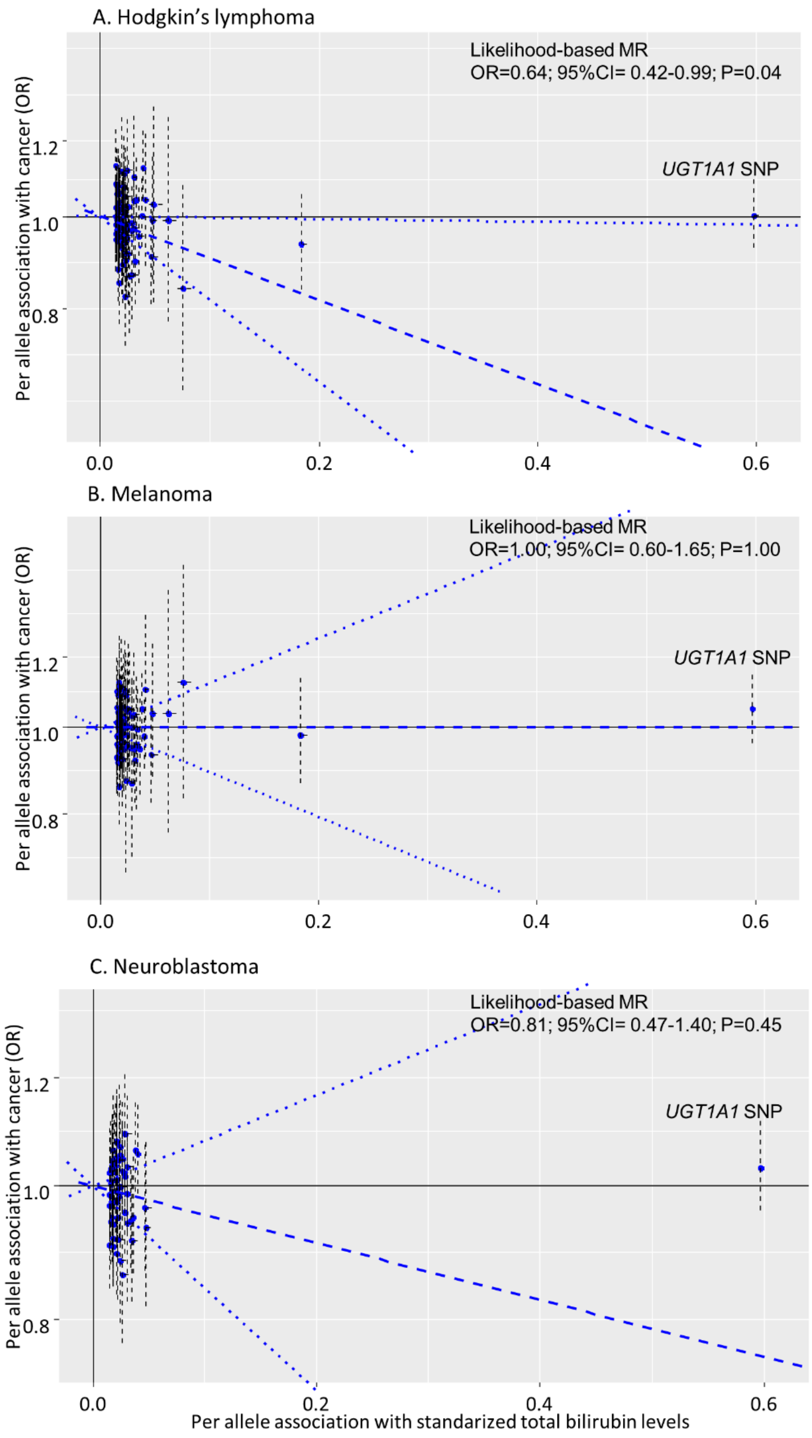

Per allele association of bilirubin SNPs with inverse-normal-transformed bilirubin levels (x axis) and risk of Hodgkin's lymphoma (A), melanoma (B), and neuroblastoma (C) (y axis; logarithmic scale), including the likelihood-based MR estimate (dashed-blue line) and its 95% confidence interval (dotted-blue lines).

Funding Sources and Acknowledgements

Genetic Consortia's specific funding sources
Funding for the genome-wide genotyping of the RCC GWAS was provided by the US National Institutes of Health (NIH), National Cancer Institute (U01CA155309) for studies coordinated by IARC and by the intramural research program of the National Cancer Institute, US NIH, for studies coordinated by the NCI.
The breast cancer genome-wide association analyses were supported by the Government of Canada through Genome Canada and the Canadian Institutes of Health Research; the 'Ministère de l'Économie; de la Science et de l'Innovation du Québec' through Genome Québec (PSR-SIIRI-701); The National Institutes of Health (U19 CA148065 and X01HG007492); Cancer Research UK (C1287/A10118, C1287/A16563, and C1287/A10710); and The European Union (HEALTH-F2-2009-223175, H2020 633784, and 634935). All studies and funders are listed in Michailidou et al.

The Prostate cancer genome-wide association analyses are supported by the Canadian Institutes of Health Research; European Commission's Seventh Framework Programme grant agreement n° 223175 (HEALTH-F2-2009-223175); Cancer Research UK Grants (C5047/A7357, C1287/A10118, C1287/A16563, C5047/A3354, C5047/A10692, and C16913/A6135); and The National Institute of Health (NIH) Cancer Post-Cancer GWAS initiative grant (1 U19 CA 148537-01) (the GAME-ON initiative).

The Prostate Cancer Program of Cancer Council Victoria also acknowledge grant support from The National Health and Medical Research Council, Australia (126402, 209057, 251533, 396414, 450104, 504700, 504702, 504715, 623204, 940394, and 614296); VicHealth; Cancer Council Victoria; The Prostate Cancer Foundation of Australia; The Whitten Foundation; PricewaterhouseCoopers; and Tattersall's. EAO, DMK, and EMK acknowledge the Intramural Program of the National Human Genome Research Institute for their support.

Genotyping of the OncoArray was funded by the US National Institutes of Health (NIH) (U19 CA 148537) for ELucidating Loci Involved in Prostate cancer SuscEptibility (ELLIPSE) project; Center for Inherited Disease Research (CIDR) (X01HG007492) under contract number (HHSN268201200008I); and by Cancer Research UK grant (A8197/A16565). Additional analytic support was provided by NIH NCI (U01 CA188392) [Schumacher].

The Breast and Prostate Cancer Cohort (BPC3) Consortium was supported by the U.S. National Institutes of Health, National Cancer Institute (cooperative agreements U01-CA98233 to D.J.H., U01-CA98710 to S.M.G., U01-CA98216 to E.R., and U01-CA98758 to B.E.H.); and Intramural Research Program of NIH/National Cancer Institute, Division of Cancer Epidemiology and Genetics.

CAPS GWAS study was supported by the Swedish Cancer Foundation (09-0677, 11-484, 12-823); the Cancer Risk Prediction Center (CRisP; www.crispcenter.org); a Linneus Centre (Contract ID 70867902) financed by the Swedish Research Council; and Swedish Research Council (K2010-70X-20430-04-3, and 2014-2269).

Prostate Cancer Genome-wide Association Study for Uncommon Susceptibility Loci (PEGASUS) was supported by the Intramural Research Program, Division of Cancer Epidemiology and Genetics, National Cancer Institute; and National Institutes of Health.

The iCOGS and OncoArray endometrial cancer analysis were supported by NHMRC project grants (ID#1031333; and ID#1109286). Funding for the iCOGS infrastructure came from: the European Community's Seventh Framework Programme (223175) (HEALTH-F2-2009-223175) [COGS]; Cancer Research UK (C1287/A10118, C1287/A 10710, C12292/A11174, C1281/A12014, C5047/A8384, C5047/A15007, C5047/A10692, and C8197/A16565); the National Institutes of Health (CA128978); and Post-Cancer GWAS initiative (1U19 CA148537, 1U19 CA148065 and 1U19 CA148112 - the GAME-ON initiative); the Department of Defence (W81XWH-10-1-0341); the Canadian Institutes of Health Research (CIHR) for the CIHR Team in Familial Risks of Breast Cancer; Komen Foundation for the Cure; the Breast Cancer Research Foundation; and the Ovarian Cancer Research Fund. OncoArray genotyping of ECAC cases was performed with the generous assistance of the Ovarian Cancer Association Consortium (OCAC). We particularly thank the efforts of Cathy Phelan. The OCAC OncoArray genotyping project was funded through grants from the US National Institutes of Health (CA1X01HG007491-01, U19-CA148112, R01-CA149429, and R01-CA058598); Canadian Institutes of Health Research (MOP-86727); and the Ovarian Cancer Research Fund. CIDR genotyping for the Oncoarray was conducted under contract 268201200008I. OncoArray genotyping of the BCAC controls was funded by Genome Canada Grant (GPH-129344, NIH Grant U19 CA148065, and Cancer UK Grant C1287/A16563).

Stage 1 and stage 2 case genotyping was supported by the NHMRC (ID#552402 and ID#1031333). Control data were generated by the Wellcome Trust Case Control Consortium (WTCCC); and a full list of the investigators who contributed to the generation of the data is available from the WTCCC website. We acknowledge use of DNA from the British 1958 Birth Cohort collection, funded by the Medical Research Council grant (G0000934); and the Wellcome Trust grant (068545/Z/02) - funding for this project was provided by the Wellcome Trust under award 085475. NSECG was supported (EU FP7 CHIBCHA), Wellcome Trust Centre for Human Genetics Core Grant (090532/Z/09Z); and CORGI was funded by Cancer Research UK. We thank Nick Martin, Dale Nyholt and Anjali Henders for access to GWAS data from QIMR Controls. Recruitment of the QIMR controls was supported by the NHMRC. The University of Newcastle; the Gladys M Brawn Senior Research Fellowship scheme; The

Vincent Fairfax Family Foundation; the Hunter Medical Research Institute; and the Hunter Area Pathology Service all contributed towards the costs of establishing the Hunter Community Study. The WHI program is funded by the National Heart, Lung, and Blood Institute; the US National Institutes of Health and the US Department of Health and Human Services (HHSN268201100046C, HHSN268201100001C, HHSN268201100002C, HHSN268201100003C, HHSN268201100004C and HHSN271201100004C). This work was also funded by NCI U19 CA148065-01. This research has been conducted using the UK Biobank Resource under applications 5122 and 9797.

ANECS recruitment was supported by project grants from the NHMRC (ID#339435); The Cancer Council Queensland (ID#4196615); and Cancer Council Tasmania (ID#403031 and ID#457636). SEARCH recruitment was funded by a programme grant from Cancer Research UK (490/A10124). The Bavarian Endometrial Cancer Study (BECS) was partly funded by the ELAN fund of the University of Erlangen. The Hannover-Jena Endometrial Cancer Study was partly supported by the Rudolf Bartling Foundation. The Leuven Endometrium Study (LES) was supported by the Verelst Foundation for endometrial cancer. The Mayo Endometrial Cancer Study (MECS) and Mayo controls (MAY) were supported by grants from the National Cancer Institute of United States Public Health Service (R01 CA122443, P30 CA15083, P50 CA136393, and GAME-ON the NCI Cancer Post-GWAS Initiative U19 CA148112), the Fred C and Katherine B Andersen Foundation; the Mayo Foundation; and the Ovarian Cancer Research Fund with support of the Smith family, in memory of Kathryn Sladek Smith. MoMaTEC received financial support from a Helse Vest Grant; the University of Bergen; Melzer Foundation; The Norwegian Cancer Society (Harald Andersens legat); and The Research Council of Norway and Haukeland University Hospital. The Newcastle Endometrial Cancer Study (NECS) acknowledges contributions from the University of Newcastle; The NBN Children's Cancer Research Group; Ms Jennie Thomas; and the Hunter Medical Research Institute. RENDOCAS was supported through the regional agreement on medical training and clinical research (ALF) between Stockholm County Council and Karolinska Institutet (20110222, 20110483, 20110141, and DF 07015); The Swedish Labor Market Insurance (100069]); and The Swedish Cancer Society (11 0439). The Cancer Hormone Replacement Epidemiology in Sweden Study (CAHRES; formerly called The Singapore and Swedish Breast/Endometrial Cancer Study; and SASBAC) was supported by funding from the Agency for Science, Technology and Research of Singapore (A*STAR); and the US National Institutes of Health and the Susan G. Komen Breast Cancer Foundation.

The Nurses' Health Study (NHS) is supported by the NCI, NIH (UM1 CA186107, P01 CA087969, R01 CA49449, 1R01 CA134958, and 2R01 CA082838). The authors thank the participants and staff of the Nurses' Health Study for their valuable contributions as well as the following state cancer registries for their help: AL, AZ, AR, CA, CO, CT, DE, FL, GA, ID, IL, IN, IA, KY, LA, ME, MD, MA, MI, NE, NH, NJ, NY, NC, ND, OH, OK, OR, PA, RI, SC, TN, TX, VA, WA, WY. The authors assume full responsibility for analyses and interpretation of these data. The authors also thank Channing Division of Network Medicine; Department of Medicine, Brigham and Women's Hospital; and Harvard Medical School. Finally, the authors also acknowledge Pati Soule and Hardeep Ranu for their laboratory assistance. The Connecticut Endometrial Cancer Study was supported by NCI, NIH (RO1CA98346). The Fred Hutchinson Cancer Research Center (FHCRC) is supported by NCI, NIH (NIH RO1 CA105212, RO1 CA 87538, RO1 CA75977, RO3 CA80636, NO1 HD23166, R35 CA39779, KO5 CA92002); and funds from the Fred Hutchinson Cancer Research Center. The Multiethnic Cohort Study (MEC) is supported by the NCI, NHI (CA54281, CA128008, and 2R01 CA082838). The California Teachers Study (CTS) is supported by NCI, NIH (2R01 CA082838, R01 CA91019, and R01 CA77398); and contract 97-10500 from the California Breast Cancer Research Fund. The Polish Endometrial Cancer Study (PECS) is supported by the Intramural Research Program of the NCI. The Prostate, Lung, Colorectal, and Ovarian Cancer Screening Trial (PLCO) is supported by the Extramural and the Intramural Research Programs of the NCI.

We would also like to thank the following for funding support: The Institute of Cancer Research and The Everyman Campaign; The Prostate Cancer Research Foundation; Prostate Research Campaign UK (now Prostate Action); The Orchid Cancer Appeal; The National Cancer Research Network UK; and The National Cancer Research Institute (NCRI) UK. We are grateful for support of National Institute for Health Research (NIHR) funding to the NIHR Biomedical Research Centre at The Institute of Cancer Research and The Royal Marsden NHS Foundation Trust.

We thank ILCCO-TRICL consortium; The PRACTICAL consortium; CRUK; BPC3; CAPS; and PEGASUS.

For the Renal Cancer Consortium:

Agricultural Health Study

This research was supported by the Intramural Research Program of the NIH, National Cancer Institute, Division of Cancer Epidemiology and Genetics (Z01CP010119).

ATBC

The ATBC Study was supported by funding provided by the Intramural Research Program of the NCI, NIH, and through U.S. Public Health Service contracts (N01-CN-45165, N01-RC-45035 and N01-RC-37004) from the NCI.

BioVU
The dataset used in the analyses described were obtained from the Vanderbilt University Medical Center resource BioVU, which is supported by institutional funding, the 1S10RR025141-01 instrumentation award, and by the Vanderbilt CTSA grant UL1 TR000445 from NCATS/NIH. Sample processing and phenotyping algorithm development was supported by institutional funding for TLE.

Center 'Bioengineering' of the Russian Academy of Sciences/Kurchatov Scientific Center
The work conducted for this study was supported by the grant Russian Scientific Fund 14-14- 01202.

Centre National de Genotypage, France
We thank Jean Guillaume Garnier and Delphine Bacq-Daian for their work on the IARC-2 scan.

ConFIRM/MCCS
The ConFIRM study, also known as CARES, was supported by the Victorian Cancer Agency (PTCB08_05), the Australian National Health and Medical Research Council (Project Grant 1011626). We acknowledge the contribution of Professor Graham Giles in supporting this work and of Ms Olive Schmid and Ms Jennifer Walsh for the project management.
The Melbourne Collaborative Cohort Study (MCCS) recruitment was funded by VicHealth and Cancer Council Victoria. The MCCS was further supported by Australian NHMRC grants 209057, 251553 and 504711 and by infrastructure provided by Cancer Council Victoria.

CPS-II
The Cancer Prevention Study II Nutrition Cohort is supported by the American Cancer Society.
The authors thank all of the men and women in the Cancer Prevention Study II Nutrition Cohort for their many years of dedicated participation in the study.

Health Professionals Follow-up Study (HPFS) and Nurses' Health Study (NHS)
The HPFS is supported by National Institutes of Health, National Cancer Institute grant UM1 CA167552. The NHS is supported by National Institutes of Health, National Cancer Institute grants UM1 CA186107 and P01 CA87969. We would like to thank the following state cancer registries for their help: AL, AZ, AR, CA, CO, CT, DE, FL, GA, ID, IL, IN, IA, KY, LA, ME, MD, MA, MI, NE, NH, NJ, NY, NC, ND, OH, OK, OR, PA, RI, SC, TN, TX, VA, WA, WY. The authors assume full responsibility for analyses and interpretation of these data.

K2 study
This study was supported by the EU FP7 under grant agreement number 241669 (the CAGEKID project). In Czech Republic, this work was also supported by MH CZ—DRO (MMCI, 00209805) and by the project MEYS – NPS I – LO1413, Czech Republic.

Leeds Cohort
The infrastructure support from Cancer Research UK as part of the Leeds Centre and
Experimental Cancer Medicine Centre funding is gratefully acknowledged together with the Leeds Multidisciplinary Research Tissue Bank, all patients who consented to take part in the research studies and the staff of the Urology and Oncology Departments in Leeds Teaching Hospitals Trust.

Mayo Clinic
This study was partially supported by National Institutes of Health: R21CA176422 (JEEP) and R01CA134466 (ASP). The authors acknowledge the Mayo Clinic Comprehensive Cancer Center Biospecimens Accessioning and Processing Shared Resource and the Pathology Research Core Shared Resource.

MD Anderson
This work was supported in part by the NIH (grant R01 CA170298) and the Center for
Translational and Public Health Genomics, Duncan Family Institute for Cancer Prevention and Risk Assessment, The University of Texas MD Anderson Cancer Center.

NCI/IARC RCC Study in Central Europe (CE)
This project was supported by the Intramural Research Program of the NIH and the National Cancer Institute.

Physicians' Health Study (PHS)
This study was supported by grants CA 097193, CA 34944, CA 40360, HL 26490, and HL 34595 from the National Institutes of Health, Bethesda, MD.

PLCO
This research was supported by the Intramural Research Program of the National Cancer Institute and by contracts from the Division of Cancer Prevention, National Cancer Institute, NIH, DHHS.

SEARCH
This study is funded by Cancer Research UK (C490/A16651).

UK GWAS
We acknowledge support from the Medical Research Council (MRC), Cancer Research UK, an educational grant from Bayer and NHS funding for the Royal Marsden Biomedical Research Centre and Cambridge University Health Partners. JL is supported by the NIHR RM/CR Biomedical Research Centre for Cancer.

US Kidney Cancer Study
The NCI United Stated Kidney Cancer Study was supported by the Intramural Research Program of the National Institutes of Health and the National Cancer Institute under the following contracts: N02-CP-10128 (Westat, Inc.), N02-CP-11004 (Wayne State University), and N02-CP- 11161 (University of Illinois at Chicago).

Van Andel Research Institute (VARI)
The authors would like to thank Dr. Kyle Furge for his role in this project as well as Drs. Anthony Avallone, John Ludlow and Philip Wise for contributing samples for this project.

Women's Health Initiative (WHI)
The WHI program is funded by the National Heart, Lung, and Blood Institute, National Institutes of Health, U.S. Department of Health and Human Services through contracts HHSN268201100046C, HHSN268201100001C, HHSN268201100002C, HHSN268201100003C, HHSN268201100004C, and HHSN271201100004C. For a list of all the investigators who have contributed to WHI science, please visit:
https://www.whi.org/researchers/Documents%20%20Write%20a%20Paper/WHI%20Investigator%20Long%20List.pdf'.

Women's Health Study (WHS)
The study is supported by grants CA-047988, HL-043851, HL-080467, HL-099355, and UM1 CA182913 from the National Institutes of Health, Bethesda, MD.
